# CYFIP1 coordinate with RNMT to induce osteosarcoma cuproptosis via AURKAIP1 m7G modification

**DOI:** 10.1186/s10020-025-01127-3

**Published:** 2025-02-21

**Authors:** Zili Lin, Ziyi Wu, Yizhe He, Xiangyao Li, Wei Luo

**Affiliations:** 1 Department of Orthopaedics, Xiangya Hospital, Central South University, Changsha, Hunan 410008 P.R. China; 2 National Clinical Research Center for Geriatric Disorders, Xiangya Hospital, Changsha, Hunan 410008 P.R. China; 3 Department of Orthopaedics, The Second Xiangya Hospital, Central South University, Changsha, Hunan 410011 P.R. China

**Keywords:** AURKAIP1, Cuproptosis, CYFIP1, FDX1, m7G modification, RNMT

## Abstract

Osteosarcoma (OS) presents challenges due to its genomic instability and complexity, necessitating investigation into its oncogenesis and progression mechanisms. Recent studies have implicated m7G, a post-transcriptional modification, in the development of various cancers. However, research on m7G modification in OS remains limited. This study aimed to explore the impact of m7G modification in OS, focusing on the role and mechanism of CYFIP1, a member of m7G cap binding complexes. Our findings demonstrated prominent anti-OS effects of CYFIP1 in vitro and vivo. Mechanistically, CYFIP1 collaborated with RNMT to induce the m7G methylation of AURKAIP1 mRNA, which resulted in the stability and the increasing translation of AURKAIP1 mRNA. AURKAIP1, a kind of mitochondrial small ribosomal subunit protein, exhibited increased expression, leading to the dysregulation of mitochondrial translation. This, in turn, caused an increase in the expression of FDX1, eventually triggering cuproptosis in OS cells and repressing OS occurrence and progression. In summary, our study identified the CYFIP1/RNMT/AURKAIP1/FDX1 axis as a potential therapeutic target for OS. These insights contribute to OS research and may guide the development of novel treatments for this challenging disease.

**Supplementary Information:**

The online version contains supplementary material available at 10.1186/s10020-025-01127-3.

## Introduction

Osteosarcoma (OS) stands as the most prevalent primary bone malignancy, predominantly affecting the adolescents(Ritter et al. 2010). Substantial progress in the realm of multidisciplinary therapies, encompassing surgical interventions, chemotherapy, radiotherapy, and targeted therapies, have notably improved the 5-year survival rates among primary OS patients while the prognosis for individuals afflicted with metastatic or recurrent OS remains disconcertingly dismal(Ritter et al. 2010; Gill and Gorlick 2021; Meltzer and Helman 2021). In actuality, a majority of OS patients present with micrometastases at the time of diagnosis, thereby placing paramount importance on the chemotherapeutic effectiveness for their survival(Gill and Gorlick 2021; Meltzer and Helman 2021). Nevertheless, the escalating emergence of chemoresistance has engendered a persistent plateau in the therapeutic effectiveness over successive decades and the adverse effects derived from chemotherapy seriously compromise the physical and psychological well-being of OS patients, necessitating a pressing quest for more efficacious and precise therapeutic alternatives(Gill and Gorlick 2021; Meltzer and Helman 2021). Regrettably, targeted therapies have encountered formidable barriers owing to OS intricate genomic intricacies and genetic instability(Wu and Livingston 2020; Kansara et al. 2014). Collectively, the limited mechanistic understanding of OS occurrence and metastasis results in the unsatisfactory therapeutic effects, highlighting the importance of profound investigation into OS pathogenesis and progression.

The profound understanding of genetics has highlighted the cancer-therapeutic role of the regulatory mechanism of gene expression(Fresquet et al. 2021; Tang et al. 2021). Among the diverse landscape of epigenetic regulations, post-transcriptional modifications, notably RNA modification, have emerged as pivotal players in numerous physiological and pathological statuses(Roundtree et al. 2017; Zhao et al. 2017). N7-methylguanosine (m7G) modification, a significant and evolutionarily conserved RNA modification, involves adding a methyl group to the seventh N position of RNA guanine(Merrick and Pavitt 2018; Luo et al. 2022; Xia et al. 2023). Functionally, m7G modification safeguards transcripts from degradation and plays essential roles in transcription elongation, RNA splicing, polyadenylation, nuclear export, and translation(Muthukrishnan et al. 1975; Shimotohno et al. 1977; Murthy et al. 1991; Pei and Shuman 2002; Konarska et al. 1984; Lindstrom et al. 2003; Drummond et al. 1985; Lewis and Izaurralde 1997; Furuichi et al. 1977). The dynamic nature of m7G modification has been correlated with the onset and progression of various diseases, with a particular emphasis on its role in cancer(Luo et al. 2022; Pandolfini et al. 2019; Tian et al. 2019; Arbour et al. 2021; Stefanska et al. 2014; Dunn et al. 2019; Chu and Shatkin 2008). To date, despite limited research on m7G modification, multiple regulators with m7G methyltransferase activities have been identified, including Methyltransferase 1, TRNA Methylguanosine / WD Repeat Domain 4 (METTL1/WDR4), Williams-Beuren Syndrome Chromosomal Region 22 / TRNA Methyltransferase Activator Subunit 11 − 2 (WBSCR22/TRMT112), RNA Guanine-7 Methyltransferase / RNA Guanine-7 Methyltransferase Activating Subunit (RNMT/RAM)(Luo et al. 2022; Xia et al. 2023; Chen et al. 2021; Alexandrov et al. 2002), which provides directions for research on m7G modification. At present, researches have revealed dynamic alterations in m7G methylation modification during OS occurrence and development. Wang et al. demonstrated the overexpression of METTL1 and WDR4 were correlated with the unfavorable prognosis of OS patients and their further mechanistic investigations unveiled that the METTL1/WDR4-mediated tRNA m7G modification enhances the proliferative, migratory, and invasive capacities of OS cells, as well as confers resistance to doxorubicin chemotherapy by modulating the translation of oncogenic mRNAs(Wang et al. 2023). Conversely, He et al. identified METTL1 as a potential tumor suppressor, elucidating its role in promoting OS ferroptosis and reducing chemoresistance through m7G methylation modification of Ferritin Heavy Chain 1 (FTH1) and pri-miR-26a(He et al. 2024). Therefore, the precise role and associated molecular regulatory mechanisms of m7G methylation modification in the pathogenesis and progression of OS warrant further exploration.

CYFIP1 (Cytoplasmic FMR1 Interacting Protein 1), as the member of the binding complexes of m7G cap, is a gene that encodes proteins regulating cytoskeletal dynamics and protein translation(Rubeis et al. 2013). Studies have found that CYFIP1 proteins coordinated with ABI1/2, BRK, NCKAP1, WAVE1/2 to form the WAVE regulatory complex (WRC), triggering actin polymerization and formation of membrane ruffles and lamellipodia(Rubeis et al. 2013; Abekhoukh et al. 2017; Marino et al. 2015). Additionally, recent studies have identified CYFIP1 involved in the formation of the CYFIP1-EIF4E-FMR1 complex that binds to the mRNA cap and mediates translational repression(Rubeis et al. 2013; Marino et al. 2015; Napoli et al. 2008). While previous research has primarily focused on the role of CYFIP1 in neuronal development and neurodevelopmental disorders like autism, schizophrenia, and epilepsy(Abekhoukh and Bardoni 2014; Bonaccorso et al. 2015), recent evidence highlights its involvement in the carcinogenesis and progression of various cancers(Teng et al. 2016a, b; Chang et al. 2018; Limaye et al. 2022; Silva et al. 2009), suggesting context-specific modulation of CYFIP1 holds promise as a therapeutic approach for cancer treatment. However, the role and regulatory mechanism of CYFIP1 in OS remain poorly understood, limiting its therapeutic potential for this disease.

In this study, we investigated the expression of CYFIP1 in OS tissues, revealing a compelling association between low CYFIP1 expression levels and poorer survival rates among OS patients. Moreover, to gain deeper insights into the functional role of CYFIP1 in OS, we conducted a series of experiments and uncovered its inhibitory effects on the proliferation and migration of OS cells, along with its ability to promote apoptosis. We also uncovered a previously unrecognized interaction between CYFIP1 and RNMT, which together modulate m7G modification of specific target molecules. Notably, we identified Aurora Kinase A Interacting Protein 1 (AURKAIP1) as a downstream target of the CYFIP1-RNMT complexes. This modification led to the increased mRNA stability and enhanced translation of AURKAIP1, a mitochondrial small ribosomal subunit protein, contributing to the perturbation of mitochondrial translation, the upregulation of Ferredoxin 1 (FDX1) protein and eventually triggering cuproptosis in OS cells. In summary, our study has uncovered the existence of the CYFIP1/RNMT/AURKAIP1/FDX1 axis, which represents a potential therapeutic target for OS. These findings significantly advance the field of OS research and have the potential to guide the development of innovative treatment modalities for this complex disease.

## Materials and methods

### Tissue specimens and ethical considerations

Ninety paraffin-embedded OS specimens and paired adjacent normal tissue samples were obtained from patients who underwent tumor resection at the Department of Orthopedics, Xiangya Hospital, Central South University. The patients’ demographic data, including sex, age, tumor histology, location, diameter, local recurrence, Enneking stage, metastasis, and prognosis, were collected from the hospital’s medical record management information system and used for statistical analysis. This study was approved by the Ethics Committee of Xiangya Hospital, Central South University with ethics approval number 2,021,101,013, and informed consent was obtained from the patients or their legal guardians.

### TCGA data acquisition and processing

The sequencing data and clinical data of 84 OS patients in TARGET-OS cohort were downloaded from The Cancer Genome Atlas Program (TCGA) website (https://www.cancer.gov/ccg/research/genome-sequencing/tcga). Patients were separated into high- and low-risk group based on the median mRNA expression and survival curves were generated using the Kaplan–Meier method.

### Cell lines, reagents and transfection

The human osteoblastic cell line hFOB1.19 and OS cell lines MG63 and U2OS were purchased from Pricella Company (Wu Han, China). Under the circumstance of 5% CO_2_ and 37 °C, MG63 and U2OS cells were cultured in Dulbecco’s modified Eagle’s medium (DMEM) (Biological Industries, Israel) while hFOB1.19 cells were maintained in DMEM/F12 medium (Pricella, Wu Han, China). Antibiotics and 10% fetal bovine serum (Gibco, USA) were added in the medium.

These antibodies used in the experiments were as follows: anti-CYFIP1 (ab108220), anti-RNMT (ab254842), anti-E-Cadherin (ab40772), anti-N-Cadherin (ab76011), anti-Caspase3 (ab32351), anti-FDX1 (ab108257), anti-Ki67 (ab15580), anti-CCND1 (ab16663), anti-DYKDDDDK (66008-4-ig, proteintech, China), anti-AURKAIP1 (821937, zenbio, China). Copper chelator, Cuprizone, (370-81-0, MCE) was purchased from MedChemExpress company.

CYFIP1-overexpressed lentivirus and negative control were purchased from Genechem Company (Shanghai, China). siRNAs and shRNAs were synthesized in General Biosystems Company (Nanjing, China). CYFIP1 stably expressed OS cells were infected with the lentivirus and selected with puromycin (1.2 mg/ml) for 4 weeks. Lipofectamine3000 (Invitrogen) was used to transfecte RNAs into tumor cells. The sequences are listed in Supplementary Table S1.

### Immunohistochemistry (IHC) and immunocytochemistry (ICC)

Tissue slides and cell slides were incubated with the corresponding primary and secondary antibodies, details of which were implemented as described in our previous studies(Yuan et al. 2022).

### RNA extraction and quantitative polymerase chain reaction (qPCR)

The total RNA was extracted using the AG RNAex Pro RNA extraction kit (AG, Changsha, China) and utilized to synthesize cDNA with the Reverse Transcription kit (AG, Changsha, China). qPCR analysis was carried out on the ABI7500 system using the TBGreen Premix Pro Taq HS Qpcr Kit (AG, Changsha, China). Lastly, we utilized the ΔΔCq method to calculate the relative expression levels of each sample and the results were expressed as 2^−ΔΔCq^. The primer sequences used for all qPCR experiments are listed in Supplementary Table S2.

### Western blotting

Total proteins were extracted from different kinds of cell lysates, separated by 10% SDS polyacrylamide gel electrophoresis, and then transferred onto the polyvinylidene membrane. Incubating in 5% defatted milk, the membranes were incubated overnight with primary antibodies at 4℃ and were then incubated with secondary antibodies for 1 h. Finally, signals of the blots were visualized using an enhanced ECL kit (BL520A, Biosharp, China).

### Cell proliferation and colony formation

CCK-8 kit (Beyotime, China) and EdU kit (RiboBio, China) were employed to assess cell proliferation. Around 5000 cells/well and 1.2 × 10^4^ cells/well were seeded in 96-well plates, respectively. The kits’ working solutions were added as per the manufacturer’s instructions. Cell proliferation was measured using a multifunctional enzyme labeling instrument and fluorescence microscope. For colony formation assay, 1000 cells were seeded in a well of 6-well plates and cultured for 2 weeks.

### Transwell migration and scratch wound-healing assay

For transwell assay, 3 × 10^4^ cells in 200 µL serum-free medium were added to upper chambers (24-well insert, Corning, USA). Bottom chambers contained 800 µL complete medium. After 24 h incubation, cells were fixed in 4% paraformaldehyde and stained with 0.1% crystal violet. Migrating cells in the bottom chamber were counted in five random fields under a microscope after scraping off cells in the upper chamber. In the scratch wound-healing assay, 3 × 10^5^ cells were seeded in 6-well plates. When cells reached 90% confluence, a scratch was made using a 1 ml tip head. After 24 h, images were captured using an inverted microscope.

### Flow cytometry

For cell apoptosis assay, cells were digested with EDTA-free trypsin, washed twice with pre-cooled PBS, and stained using an apoptosis detection kit (#FXP023, 4 A BIOTECH, China). Subsequently, the stained samples were analyzed by flow cytometry after incubation at room temperature in the dark for 10 min.

For reactive oxygen species (ROS) analysis, cells were seeded in 6-well plates and were stained with DHE (US Everbright, Suzhou) diluted with Hank’s solution (ratio 1:1000) in an incubator at 37 °C for 20 min. Then, cells were digested with trypsin and washed twice with pre-cooled PBS. Finally, the stained samples were detected by flow cytometry.

For mitochondrial membrane potential (MMP), cells were seeded in 6-well plates and were stained with TMRM (US Everbright, Suzhou) diluted with Hank’s solution (ratio 1:2000) in an incubator at 37 °C for 20 min. Then, cells were digested with trypsin and washed twice with pre-cooled PBS. Finally, the stained samples were detected by flow cytometry.

### RNA stability assay

OS cells were seeded in 6-well plates overnight, and then treated with 5 µg/mL actinomycin D (#S8964, Selleck, USA) at the 0, 3, 6 h. Total RNA was then extracted using TRIzol reagent (AG, Changsha, China) and analyzed by qPCR. Eventually, the linear regression analysis was utilized to estimate the mRNA half-lives time.

### RNA-sequencing

The total RNA was extracted from CYFIP1 stably expressed U2OS cells and the corresponding control cells using TRIzol reagent (AG, Changsha, China) according to the manufacturer’s protocol. The RNA sample quantification, qualification, library preparation, transcriptome sequencing and subsequent analysis were conducted by OE Biotech Co., Ltd. (Shanghai, China). Differential expression analysis was performed using the DESeq2(Love et al. 2014). Q value < 0.05 and foldchange > 2 or foldchange < 0.5 was set as the threshold for significantly differential expression gene (DEGs).

### Mass spectrum and CO-IP

Cell lysis was performed using Pierce™ IP Lysis Buffer (XG351119, Thermo scientific, USA) according to the manufacturer’s instructions. Next, the supernatant was extracted by high-speed centrifugation, incubated with 5 µg of specific antibodies against CYFIP1 and rotated with magnetic beads overnight at 4 °C. For mass spectrum, the protein-beads complexes derived from U2OS cell lysis were washed 3 times and proteins were eluted by 20ul glycine-HCL solution. The mass spectrum and subsequent data analysis were constructed by Novogene Corporation (Beijing, China). For CO-IP, protein-beads complexes mixed with loading buffer were incubated in 100℃ 5 min and the supernatant was extracted by high-speed centrifugation for western blotting.

### RNA immunoprecipitation (RIP)-sequencing or qPCR

RIP was conducted with the RNA immunoprecipitation Kit (BersinBio, Guangzhou, China) according to the manufacturer’s instructions. In brief, 5 µg of specific antibodies against rabbit immunoglobulin G, CYFIP1 or RNMT were incubated with prepared CYFIP1 stably expressed U2OS cells and the corresponding control cells lysates overnight at 4 °C and rotated with magnetic beads 1 h at the second day. Subsequently, the RNA-protein-beads complexes were washed 5 times and eluted by elution fluid mixed with proteinase K. Finally, phenol-chloroform-isopentanol mixture was utilized for RNA extraction. The sequencing library and subsequent data analysis were constructed by Novogene Corporation (Beijing, China). The relative interaction between CYFIP1-RNMT complexes and their targeted transcripts were validated by qPCR and normalized to the input.

### m7G meRIP-sequencing or qPCR

MeRIP experiment and high through-put sequencing and data analysis were conducted by Seqhealth Technology Co., LTD (Wuhan, China). Total RNAs were extracted from CYFIP1 stably expressed U2OS cells and the corresponding control cells and then undergone DNA digestion, quality testing, quantification. 50 µg total RNAs were used for polyadenylated RNA enrichment by VAHTS mRNA Capture Beads (VAHTS, cat. NO. N401-01/02). 20mM ZnCl2 was added to mRNA and incubated at 95℃ for 5–10 min until the RNA fragments were mainly distributed in 100-200nt. Then 10% RNA fragments was saved as “Input” and the rest was used for m7G immunoprecipitation. The specific anti-m7G antibody (#RN017M, MBL) was applied for m7G immunoprecipitation. RNA samples of both input and IP were prepared using TRIzol reagent (Invitrogen, cat. NO 15596026). After the stranded RNA sequencing using Illumina^®^ platform, raw sequencing data undergone quality control, mapping, peak annotation, and subsequent data analysis. The m7G-modified transcripts were validated by qPCR and normalized to the input.

### Mitochondrial proteomics

Mitochondrion were extracted using the mitochondrial extraction kit (SM0020, Solarbio, Beijing). Subsequently, mitochondrial proteins were extracted from cell lysates, quantified using BCA method, and separated by 12% SDS polyacrylamide gel electrophoresis. Then, the obtained proteins were undergone enzymolysis, label, enrichment, and Mass spectrometry analysis. Spectronaut (Version 15.3.210906.50606) was used to search all of the raw data thoroughly against the sample protein database. Database search was performed with Trypsin digestion specificity. Alkylation on cysteine was considered as fixed modifications in the database searching. Protein, peptide and PSM’s false discovery rate (FDR) all set to 0.01. For DIA data, the quantification FDR also set to 0.05. Quantity MS-level was set at MS2. The mass spectrum and subsequent data analysis were conducted by OE Biotech Co., Ltd. (Shanghai, China).

### Xenograft mouse models

The animal experiments in this study obtained approval from the Animal Care and Ethics Committee of Xiangya Hospital of Central South University (Changsha, China). The tumor in mice did not exceed 20 cubic mm and any dimension was less than 20 mm in diameter, which met the animal ethical standards of Xiangya Hospital. In brief, four- to six-week-old female nude mice were divided into two group containing six mice, of which one group was injected CYFIP1 stably expressed U2OS cells while other group was injected control U2OS cells. Subsequently, the tumor volume of mice was measured every 5 days, and tumors were harvested after 20 days, with their weights measured.

### Statistical analysis

GraphPad Prism 8 were used for data processing and statistical analysis of all the experimental results. The quantitative data were expressed as the mean ± SD and tested by student’s t-test was while the qualitative data expressed in frequency were analyzed by Chi-square test or Fisher’s exact test. The indicated P value (* *P* < 0.05, **P < 0.01, ***P < 0.001, and ****P < 0.0001) was considered statistically significant.

**Table 1:** Correlation of CYFIP1 expression with clinicopathological features of OS patients

Clinicopathological parameter	CYFIP1 protein expression		*P* value
	Low (*n* = 48)	High (*n* = 42)	
Age			0.9999
< 21	40	35	
≥ 21	8	7	
Sex			0.9757
Female	15	13	
Male	33	29	
Tumour size (cm)			0.0001
< 8	16	31	
≥ 8	32	11	
Local recurrence			0.1981
Yes	6	2	
No	42	40	
Enneking’s stage			< 0.0001
IIA	8	5	
IIB	20	33	
III	22	2	
Anatomic location			0.1163
Femur	24	18	
Tibia	8	16	
Humerus	11	6	
Others	5	2	
Lung metastasis			0.0072
Yes	22	8	
No	26	34	
Histology			0.2357
Conventional osteosarcoma	35	35	
Others	13	7	

Differences between groups were done by the Chi-square test

**Fig. 1:**
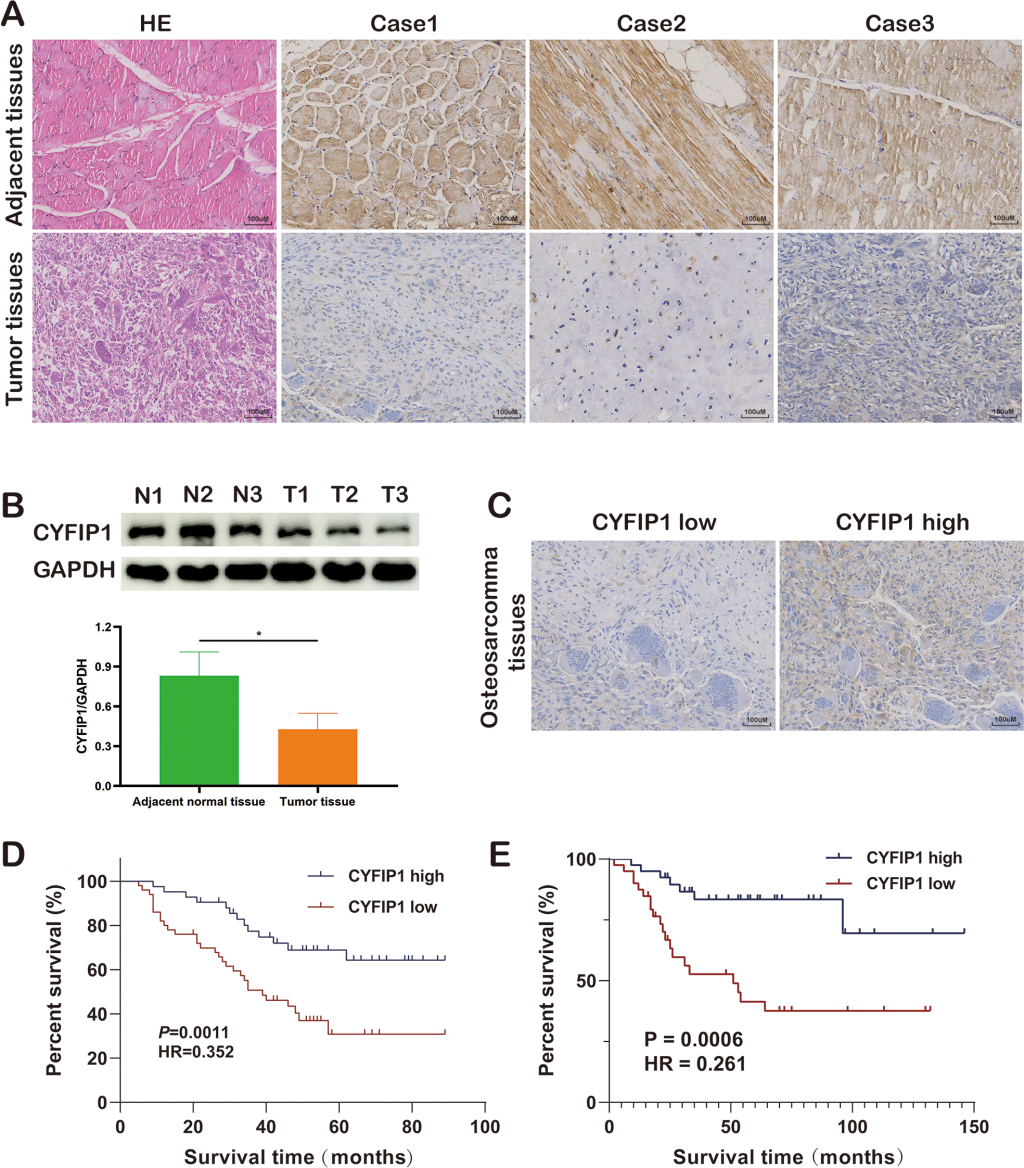
CYFIP1 expression in osteosarcoma and its relationship to patient survival. **A**,** B**. Low CYFIP1 expression was detected in OS tissues while high CYFIP1 expression in their adjacent tissues. **C**. IHC staining showed the representative images of low or high expression of CYFIP1. **D**. Kaplan-Meier survival curve exhibited that low CYFIP1 expression was associated with the worse survival statuses in Xiangya hospital (Log-rank *p* = 0.0011, HR = 0.377, n[high] = 42, n[low] = 48 ). **E**. Kaplan-Meier survival curve exhibited that low CYFIP1 expression was associated with the worse survival statuses in TARGET-OS cohort (Log-rank *p* = 0.0006, HR = 0.261, *n* = 42 per group). The data represent the mean ± S.D. *P < 0.05 or **P < 0.01 indicates a significant difference between the indicated groups

**Fig. 2:**
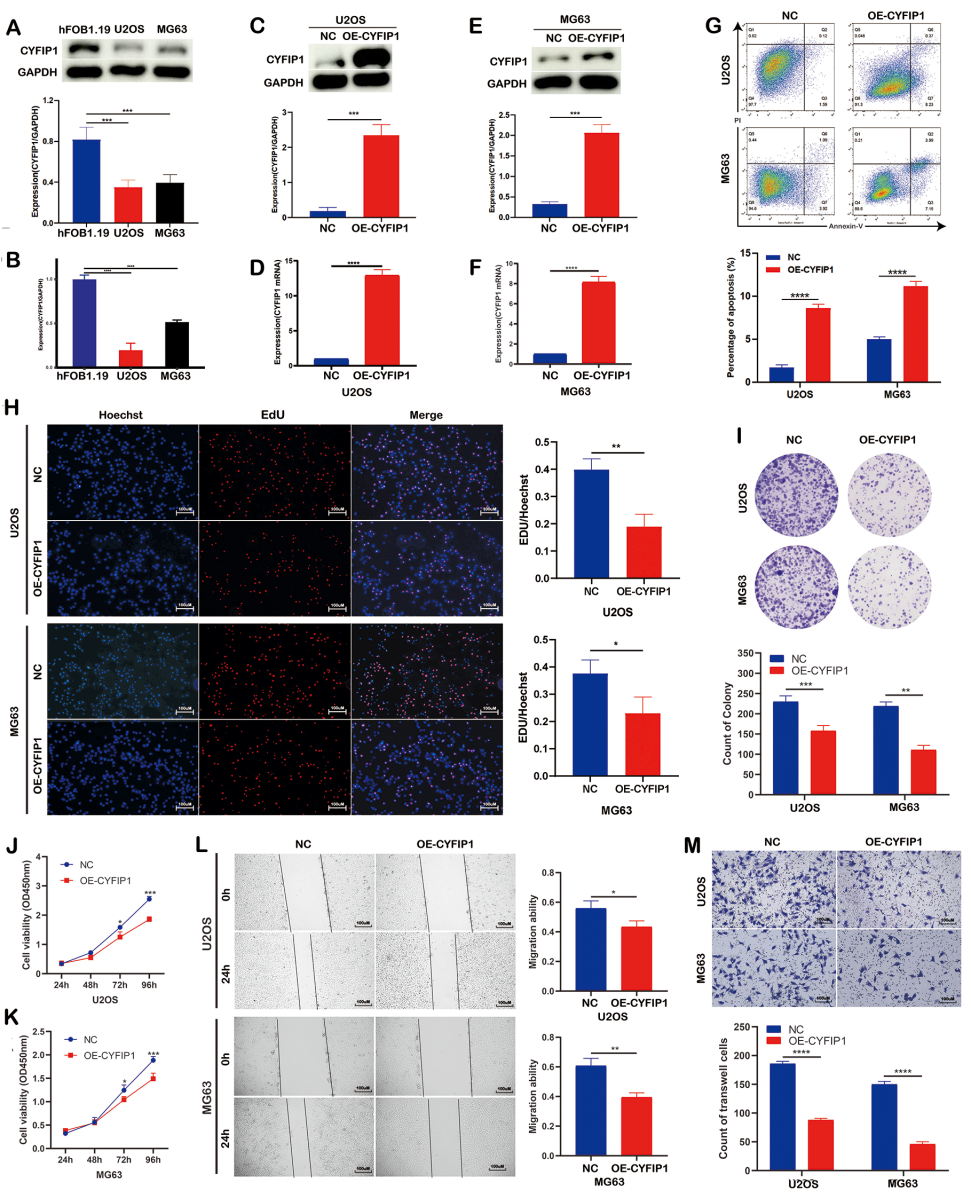
Overexpression of CYFIP1 inhibited the proliferation and metastasis while promoted the apoptosis of osteosarcoma cells in vitro. **A**,** B**. WB and qPCR exhibited the low expression of CYFIP1 in OS cells. **C-F**. WB and qPCR exhibited the overexpression efficiency of lentivirus transfection. **G**. Overexpression of CYFIP1 facilitated osteosarcoma cells apoptosis. **H-K**. CCK8. EdU and colony formation assays exhibited overexpression of CYFIP1 inhibited osteosarcoma cells proliferation. **L**,** M**. Overexpression of CYFIP1 inhibited osteosarcoma cells migration. The data represent the mean ± S.D. *P < 0.05, **P < 0.01, ***P < 0.001, and ****P < 0.0001 indicates a significant difference between the indicated groups

## Results

### Low expression of CYFIP1 was detected in OS tissues and associated with the worse survival rate

Previous studies have implicated dysregulated CYFIP1 expression in certain cancers, particularly with regards to disease progression, including invasion and metastasis(Silva et al. 2009). In our investigation, to explore the expression landscape of CYFIP1 in OS, IHC and WB assays were employed. As expected, diminished CYFIP1 expression was detected in OS tissues compared to corresponding adjacent normal tissue (Fig. 1A.B). The detailed clinicopathological features of OS patients were shown in Table 1. Further, to elucidate the association between CYFIP1 expression and overall survival rates in OS patients, we stratified them into high-expressing (*n* = 42) and low-expressing (*n* = 48) groups based on IHC staining results (Fig. 1C), and Kaplan-Meier analysis demonstrated a significant negative correlation between CYFIP1 expression and OS patients’ overall survival rate (Fig. 1D). Similarly, the negative correlation was further validated in the TARGET-OS cohort through sequencing and follow-up data (Fig. 1E). Given the differential expression of CYFIP1 between OS tissues and their corresponding adjacent normal tissues, we dedicated to explore its effect and underlying mechanism during OS pathogenesis and progression.

**Fig. 3:**
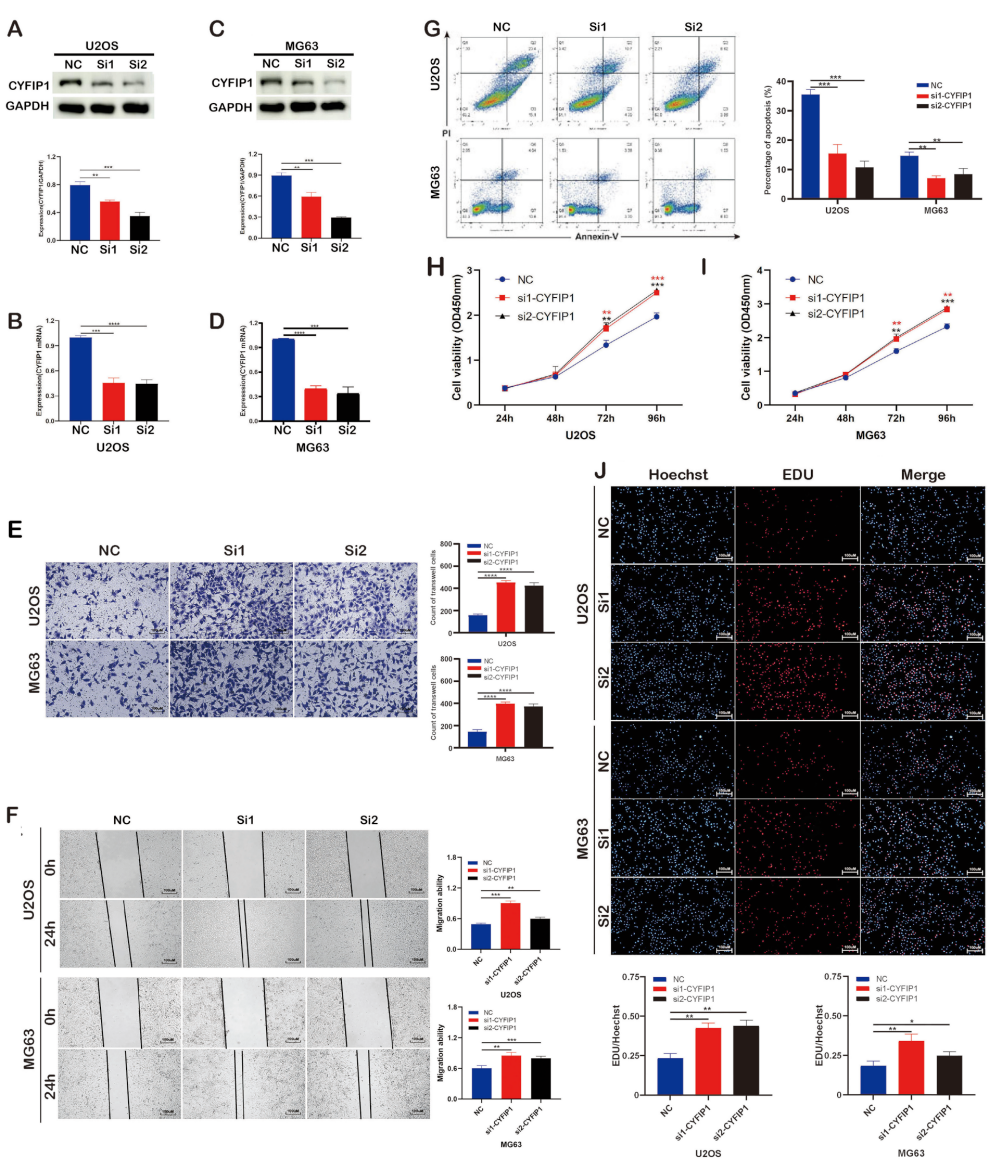
Validation of the role of CYFIP1 via downregulation experiment. **A-D**. WB and qPCR exhibited the knockdown efficiency of shRNA transfection. **E-F**. Knockdown of CYFIP1 promoted osteosarcoma cells migration. **G**. Knockdown of CYFIP1 decreased osteosarcoma cells apoptosis. **H-J**. CCK8 and EdU assays exhibited knockdown of CYFIP1 promoted osteosarcoma cells proliferation. The data represent the mean ± S.D. *P < 0.05, **P < 0.01, ***P < 0.001, and ****P < 0.0001 indicates a significant difference between the indicated groups

**Fig. 4:**
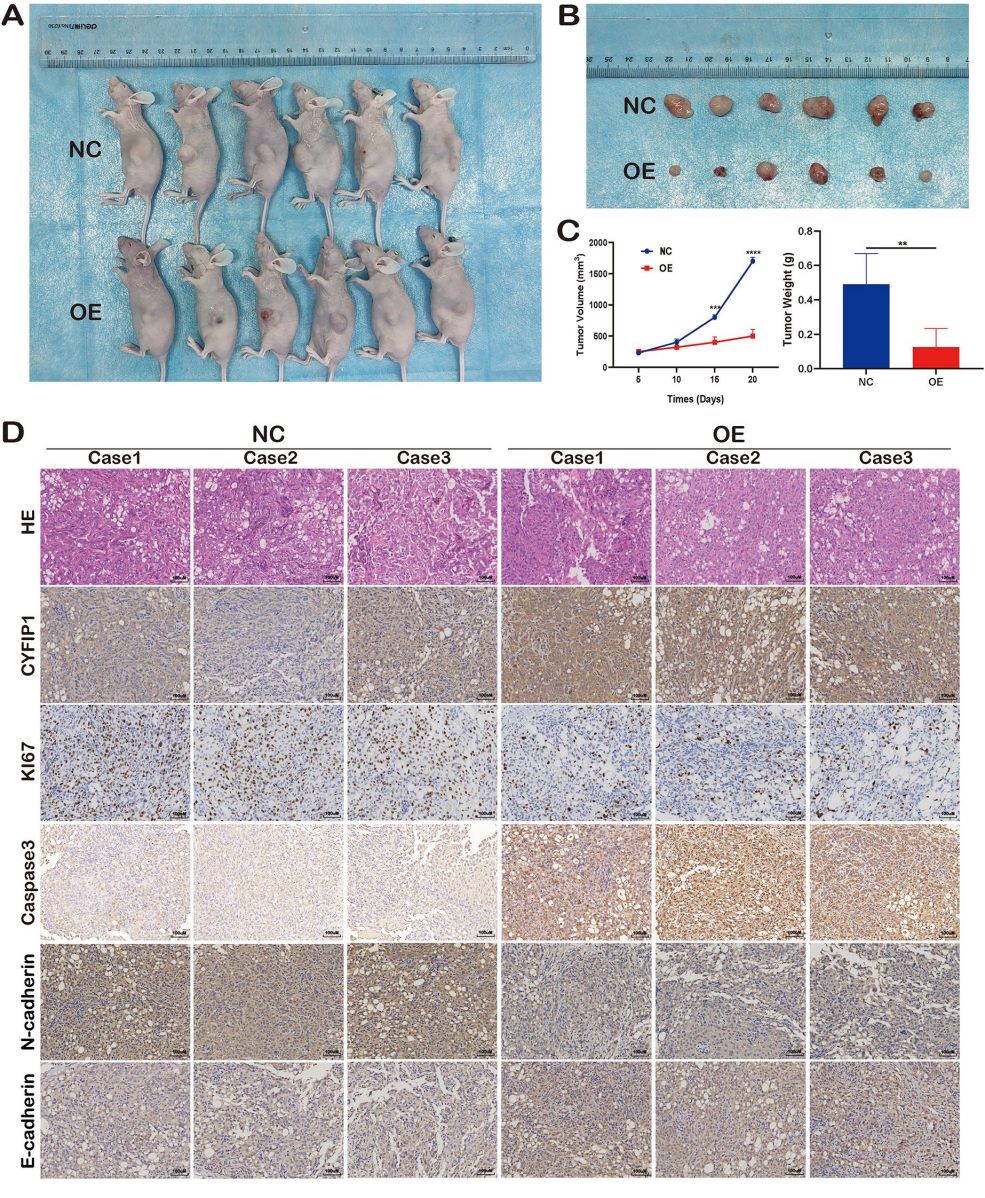
Validation of the role of CYFIP1 via in-vivo experiment. **A-C**. Overexpression of CYFIP1 inhibited osteosarcoma proliferation in vivo. **D**. Overexpression of CYFIP1 elevated the expression of E-cadhesion and caspase-3 while decreased the expression of Ki67 and N-cadhesion. The data represent the mean ± S.D. *P < 0.05, **P < 0.01, ***P < 0.001, and ****P < 0.0001 indicates a significant difference between the indicated groups

### Over-expression of CYFIP1 suppressed the proliferation and migration and facilitated the apoptosis of OS cells

To validate CYFIP1 expression at the cytological level, we analyzed CYFIP1 proteins and RNAs extracted from OS cells and osteoblasts. The results revealed a downregulation of both CYFIP1 mRNAs and proteins in OS cells, which was consistent with our histological experiment (Fig. 2A.B). Subsequently, we sought to investigate the impact of CYFIP1 on the biological behavior of OS cells by enhancing CYFIP1 expression through lentivirus transfection (Fig. 2C-F). Following the upregulation of CYFIP1 expression, we observed a decrease in cell viability through colony formation, CCK8, and EdU assays, along with a substantial increase in OS cell apoptosis (Fig. 2G-K). Considering previous studies indicating CYFIP1 as a suppressor of invasion in various cancers, we also explored its role in OS metastasis and found that overexpression of CYFIP1 significantly inhibited the migration of OS cells, which corroborated earlier findings (Fig. 2L.M). To further investigate the impact of CYFIP1, we employed siRNA to knock down CYFIP1 expression and confirmed its downregulation using WB and qPCR (Fig. 3A-D). Consistent with the findings from CYFIP1 overexpression, the downregulation of CYFIP1 promoted the proliferation and migration of OS cells while reducing apoptosis (Fig. 3E-J). Moreover, we investigated molecules associated with apoptosis, cell cycles, and metastasis to further validate our findings. As expected, Cleaved Caspase-3 and E-cadherin were upregulated, while CCND1 and N-cadherin exhibited opposing trends (supplementary Fig. 1A.B). Therefore, CYFIP1 exhibited an excellent anti-OS effect in term of the in-vitro experiment, triggering our interesting to further exploration.

**Fig. 5:**
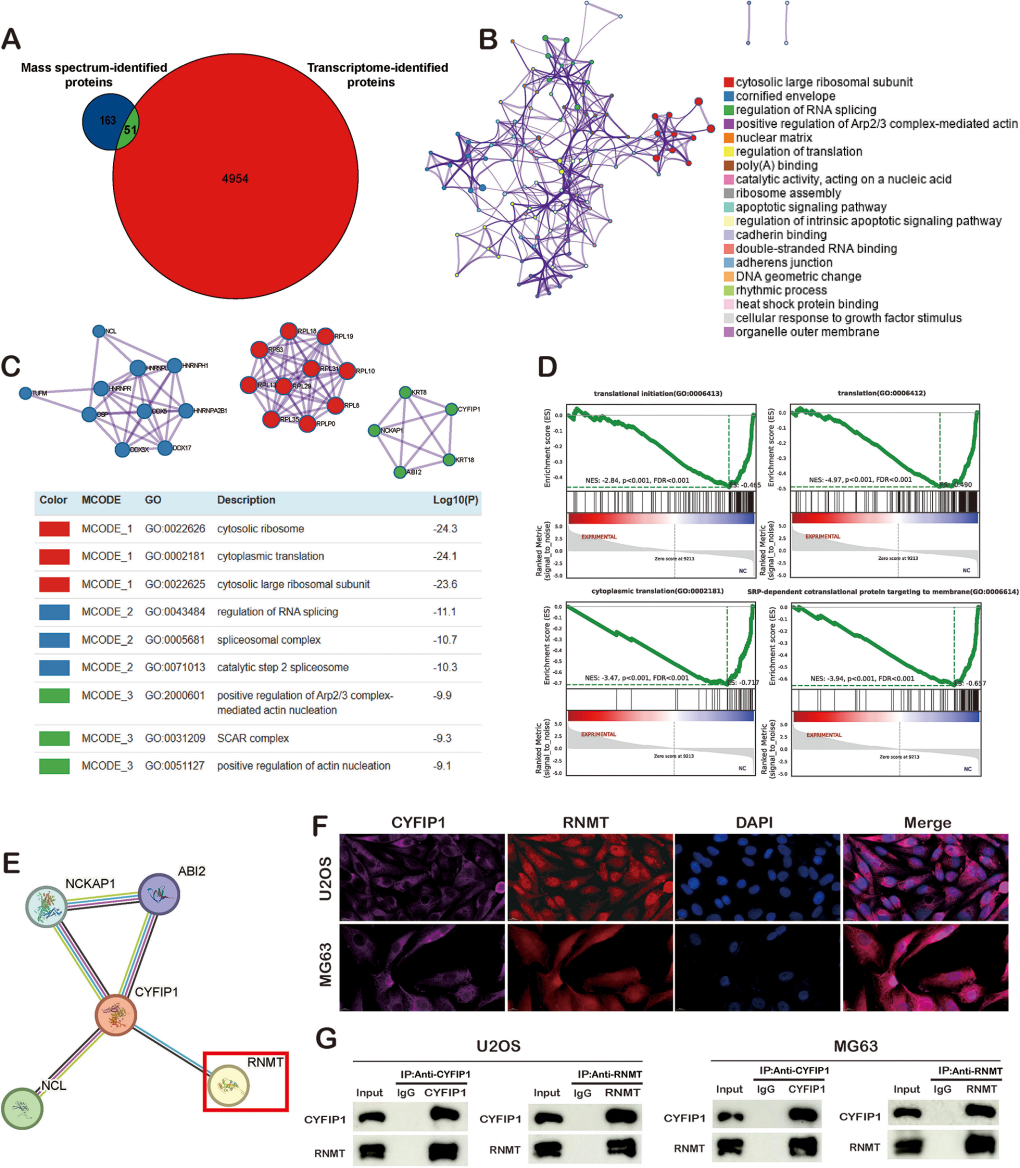
RNMT serves as a partner of CYFIP1. **A-D**. The interacted genes of CYFIP1 in mass spectrum and the corresponding functional enrichment. **E.** PPI from String website. **F-G**. ICC exhibit the localization of CYFIP1 and RNMT in osteosarcoma cells and coIP proved the interaction between CYFIP1 and RNMT

**Fig. 6:**
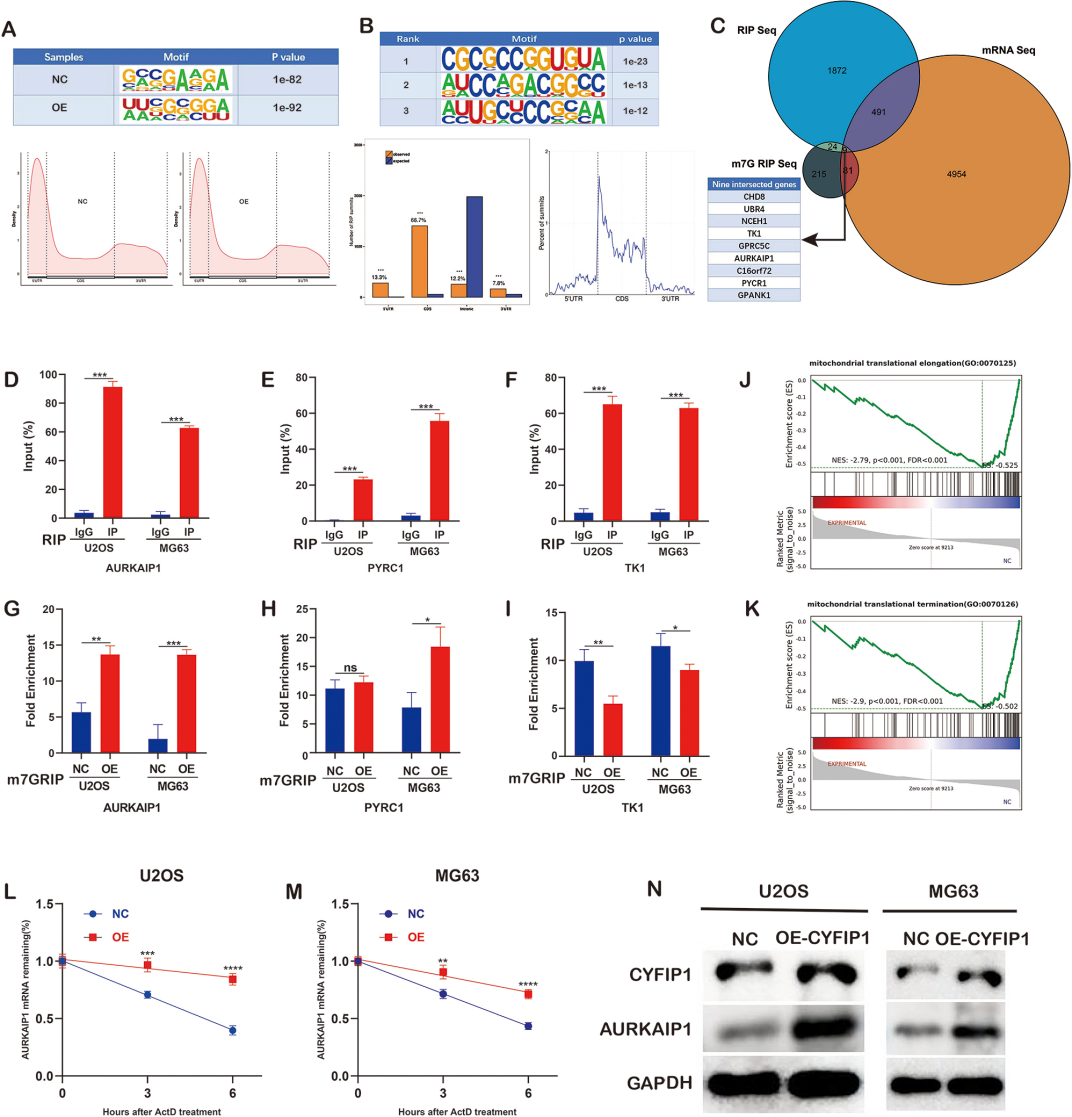
CYFIP1 coordinated with RNMT to facilitate the m7G modification of their targets. **A**,** B**. RIP and m7G-RIP sequencing. **C**. The intersected genes between mRNA, RIP and m7G-RIP sequencing. **D-I**. RIP- and m7G-RIP-qPCR identified AURKAIP1, PYRC1 and TK1 as the targets of CYFIP1-RNMT complexes. **J**,** K**. Gene set enrichment analysis of mRNA sequencing. **L**,** M**. The RNA stability assays of AURKAIP1. **N**. Overexpression of CYFIP1 increased the expression of AURKAIP1. The data represent the mean ± S.D. *P < 0.05, **P < 0.01, and ***P < 0.0001 indicates a significant difference between the indicated groups

### Overexpression of CYFIP1 suppressed human OS growth in vivo

To further investigate the tumorigenicity of CYFIP1 in vivo, we subcutaneously injected U2OS cells expressing different levels of CYFIP1 into nude mice. As anticipated, the growth of tumors derived from CYFIP1-stably-expressed OS cells was notably suppressed compared to NC group (Fig. 4A). On day 20 post-injection, the tumors were surgically excised, and their weights were measured, demonstrating the significant anti-OS effect of CYFIP1 (Fig. 4B.C). Additionally, IHC staining was performed on the tumor samples, confirming the overexpression of CYFIP1 in the CYFIP1-stably-expressed OS cells. Furthermore, compared to the NC groups, the expression of Caspase3 and E-cadherin increased, while Ki67 and N-cadherin decreased in the CYFIP1-stably-expressed groups (Fig. 4D). Overall, our in vivo assays align with the observations made in vitro, providing further evidence for the tumor-suppressing role of CYFIP1.

**Fig. 7:**
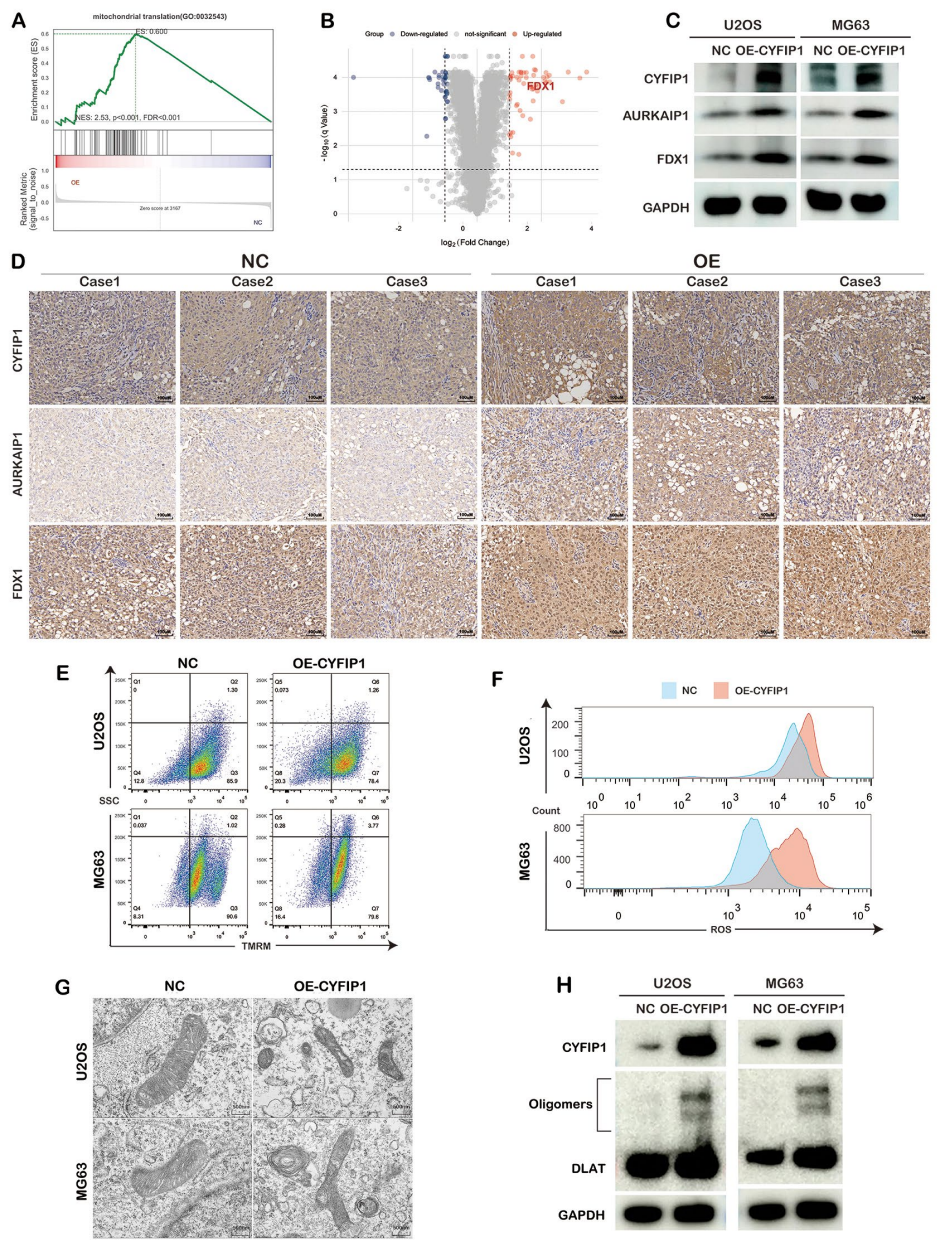
CYFIP1 coordinated with RNMT to induce cuproptosis via AURKAIP1/FDX1 pathway. **A**,** B**. Gene set enrichment analysis and volcano map of mitochondrial proteomics. **C**,** D**. Overexpression of CYFIP1 increased the expression of AURKAIP1 and FDX1. **E**. Overexpression of CYFIP1 decreased the mitochondrial membrane potential in osteosarcoma cells. **F**. Overexpression of CYFIP1 elevated the ROS level in osteosarcoma cells. **G**. Overexpression of CYFIP1 destroyed the structure of mitochondrion. **H**. The overexpression of CYFIP1 could induce the aggregation of DLAT

**Fig. 8:**
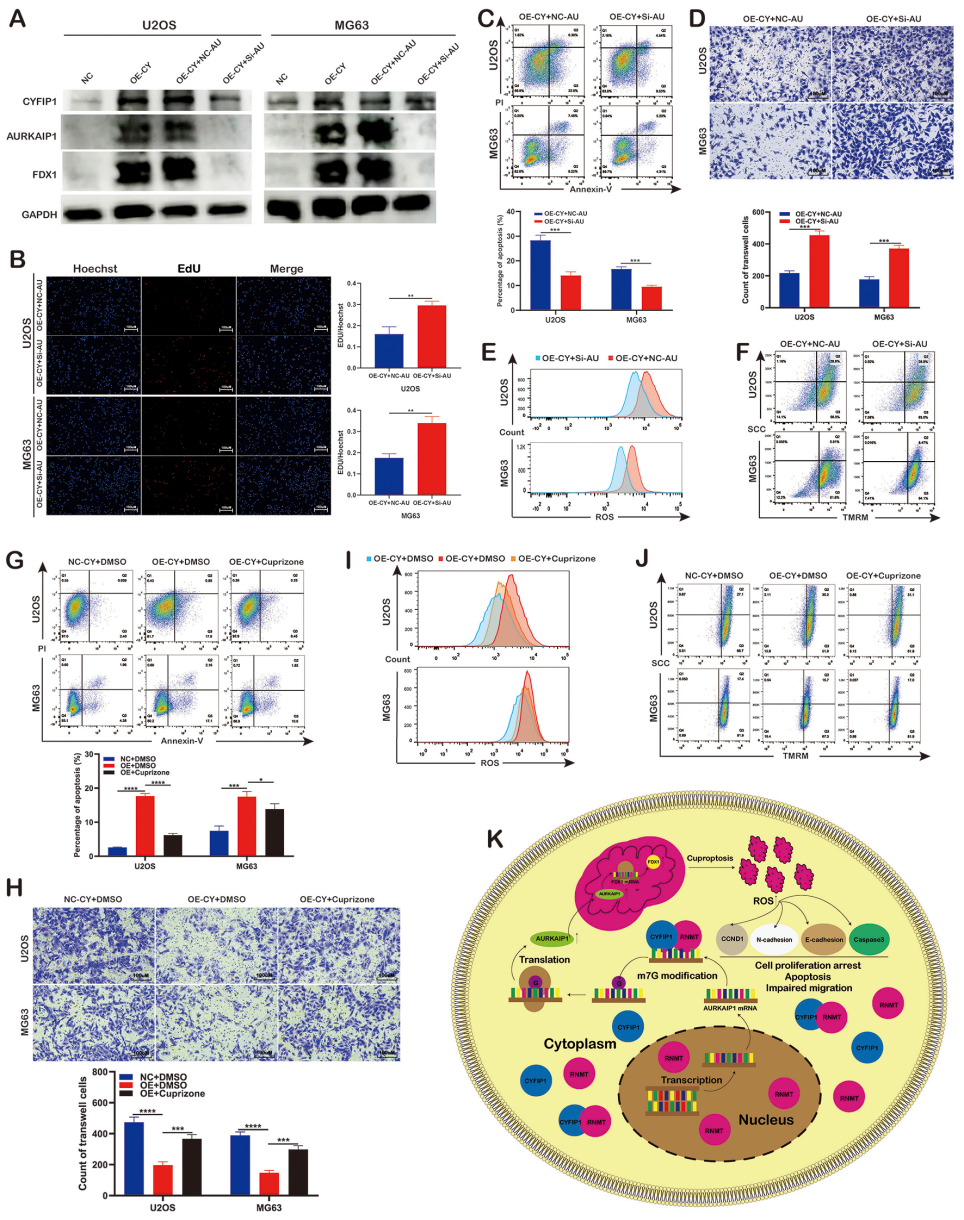
AURKAIP1 knockdown and copper chelator reversed the anti-osteosarcoma effects derived from CYFIP1 overexpression. **A**. WB exhibited the knockdown efficiency of siRNA transfection. **B**. EdU assays exhibited knockdown of AURKAIP1 rescued osteosarcoma cells proliferative ability. **C**. Knockdown of AURKAIP1 rescued osteosarcoma cells apoptosis. **D**. Knockdown of AURKAIP1 promoted osteosarcoma cells migration. **E**,** F**. Knockdown of AURKAIP1 decreased ROS level and elevated MMP. **G**. Application of copper chelator rescued osteosarcoma cells apoptosis. **H**. Application of copper chelator promoted osteosarcoma cells migration. **I**,** J**. Application of copper chelator decreased ROS level and elevated MMP. **K**. The diagram of anti-OS effects of CYFIP1. The data represent the mean ± S.D. *P < 0.05, **P < 0.01, ***P < 0.001, and ****P < 0.0001 indicates a significant difference between the indicated groups

### RNMT served as a partner of CYFIP1

To investigate the underlying mechanism of CYFIP1-mediated suppression of OS growth and metastasis, we conducted an in-depth analysis using a combination of molecular techniques and bioinformatics tools. Our study employed mRNA sequencing between CYFIP1 stably expressed groups and NC groups, and identified a total of 4954 DEGs, in which 1386 were found to be down-regulated and 3568 showed up-regulation (supplementary Fig. 2A). Subsequently, we performed comprehensive Gene Ontology (GO) and Kyoto Encyclopedia of Genes and Genomes (KEGG) analyses to gain insights into the functional implications of these DEGs. The GO analysis revealed a significant enrichment of DEGs in cytoplasmic translation-related pathways, particularly mitochondrial translation-related processes. Specifically, these pathways included translation, SRP-dependent cotranslational protein targeting to the membrane, mitochondrial translational elongation, mitochondrial translational termination, and structural constituents of ribosomes (supplementary Fig. 2B). This suggests a potential involvement of CYFIP1 in translation processes and mitochondrial metabolism. In the KEGG analysis, we observed a prominent enrichment of DEGs in pathways such as ribosome, ubiquitin-mediated proteolysis, signaling pathways regulating pluripotency of stem cells, and colorectal cancer (supplementary Fig. 2C). These findings suggest that CYFIP1 may play a regulatory role in translation-related pathways that are implicated in the pathogenesis and progression of tumors.

To further explore the interacting partners of CYFIP1, we employed mass spectrometry to identify proteins that interact with CYFIP1. Our analysis identified a set of 214 CYFIP1-interacting proteins, with a predominant representation of cytoplasmic proteins (supplementary Fig. 2D). Functional enrichment analysis of these genes using GO, KEGG, Clusters of Orthologous Groups of proteins (COGs), and InterPro (IPR) indicated a strong focus on translation-related pathways, posttranslational modification, and the RNA recognition motif domain (supplementary Fig. 2E-I). To gain more insight into the specific interactions involving CYFIP1, we intersected the DEGs with the genes corresponding to CYFIP1-interacting proteins and identified 51 genes of interest (Fig. 5A). Then, functional enrichment was performed for these 51 genes and indicated the translation-related pathways and RNA modification were enriched (Fig. 5B.C). Meantime, gene set enrichment analysis (GSEA) exhibited the translation-related pathways, particularly cytoplasmic translation, were enriched in NC groups (Fig. 5D). Subsequently, we constructed a protein-protein interaction (PPI) network using the STRING website and identified four genes—NCKAP1, ABI2, NCL, and RNMT—that exhibited significant associations with CYFIP1 (Fig. 5E). Previous studies indicated RNMT, a RNA guanine-7 methyltransferase, could take part in mRNA cap methylation and the methylation of cytoplasmically recapped RNAs, thereby influencing transcription and translation processes(Xiao et al. 2023). Considering the prevalence of translation-related pathways in the GSEA results of our transcriptome data and the co-expression relationship between CYFIP1 and RNMT in the PPI network, we hypothesize that CYFIP1 may interact with RNMT to modulate the expression and fate of specific genes. To validate the interaction between CYFIP1 and RNMT, we conducted ICC and CO-IP experiments. Consistent with our expectations, ICC analysis demonstrated that CYFIP1 primarily localizes to the cytoplasm, while RNMT exhibits both nuclear and cytoplasmic localization patterns (Fig. 5F). Furthermore, CO-IP experiments confirmed the physical interaction between CYFIP1 and RNMT (Fig. 5G). These findings strongly support the notion that RNMT may act as a functional partner of CYFIP1, contributing to its regulatory activities and biological functions.

### CYFIP1 coordinated with RNMT to m7G modify their targets and regulate their expression

Considering the identification of RNMT as an RNA guanine-7 methyltransferase, we hypothesized that CYFIP1-RNMT complexes could potentially influence the m7G modification of specific genes. To investigate this hypothesis, we performed RIP-sequencing and m7G-RIP-sequencing to identify the target genes. The results revealed 215 differential peaks in m7G-RIP-sequencing and 1872 differential peaks in RIP-sequencing (Fig. 6A-C). After annotating these peaks, we intersected them with the DEGs identified in mRNA sequencing, resulting in the identification of nine genes that were present in all three datasets: CHD8, UBR4, NCEH1, TK1, GPRC5C, AURKAIP1, C16orf72, PYCR1, and GPANK1 (Fig. 6C). To validate the sequencing results and eliminate false positives, we conducted RIP-qPCR and m7G-RIP-qPCR experiments, ultimately confirming three valid genes: AURKAIP1, TK1, and PYRC1 (Fig. 6D-I).Interestingly, GSEA of our transcriptome sequencing data revealed significant enrichment of mitochondrial metabolism-related pathways, such as mitochondrial translation elongation and mitochondrial translation termination, in the NC group (Fig. 6J.K). This finding suggests that the regulation of mitochondrial metabolism-related pathways may play a crucial role in the tumor-suppressing effect of CYFIP1. Additionally, we found that AURKAIP1, the most significant gene identified in the m7G-RIP-sequencing, is involved in mitochondrial translation-related pathways, leading us to select AURKAIP1 for further investigation (Fig. 6D.G). To validate the impact of m7G modification on the expression of AURKAIP1, we conducted an RNA stability experiment and observed that overexpression of CYFIP1 significantly extended the half-life of AURKAIP1 mRNA (Fig. 6L.M). Furthermore, through Western blot assay, we discovered that overexpression of CYFIP1 increased the expression of AURKAIP1 (Fig. 6N). Eventually, to validate the m7G modification of AURKAIP1 was derived from RNMT during CYFIP1 overexpression, we knocked down the RNMT expression and performed Western blot, RIP-qPCR and m7G-RIP-qPCR assays and found that the knock-down of RNMT indeed decreased the m7G modification and expression of AURKAIP1, supporting CYFIP1 could coordinate with RNMT to m7G modifies AURKAIP1 and regulate its expression (Supplementary Fig. 3A-C). Therefore, it can be concluded that CYFIP1 coordinate with RNMT to enhance the stability of AURKAIP1 mRNA through m7G modification, thereby facilitating the expression of AURKAIP1.

### CYFIP1 interacted with RNMT to induce OS cuproptosis via AURKAIP1/FDX1 pathway

Given the mitochondrial translation-related role of AURKAIP1 and the enrichment of mitochondrial translation-related pathways in our bioinformatics data, we performed mitochondrial proteomics to explore the downstream of AURKAIP1. Interesting, different from mRNA sequencing, mitochondrial proteomics indicated that mitochondrial translation was enriched in CYFIP1 overexpressed group (Fig. 7A).

Notably, FDX1, the core member of cuproptosis, were upregulated in the CYFIP1 overexpressed group in mitochondrial proteomics (Fig. 7B). Subsequently, to confirm the results of mitochondrial proteomics, we utilized WB blotting and found that after the overexpression of CYFIP1, both AURKAIP1 and FDX1 were upregulated (Fig. 7C). Meantime, the corresponding IHC staining slides derived from our xenograft mouse models shown that the overexpression of CYFIP1 was followed with the significant upregulation of AURKAIP1 and FDX1 (Fig. 7D). Since FDX1 was proved to result in cuproptosis and the subsequent mitochondrial dysregulation, we detected the change of MMP and ROS level after the overexpression of CYFIP1. As expected, the overexpression of CYFIP1 triggered MMP decline increased ROS level (Fig. 7E.F). Meantime, through transmission electron microscope (TEM), we found the structure of mitochondrion became disorganized after the overexpression of CYFIP1 (Fig. 7G). Since cuproptosis involves the aggregation of lipoylated proteins, we tested the aggregation of DLAT, a kind of lipoylated proteins of Tricarboxylic acid (TCA) cycle and the results of WB suggested that the overexpression of CYFIP1 could induce the aggregation of DLAT, further confirming the occurrence of OS cells cuproptosis (Fig. 7H).

To further confirmed the anti-OS effects of CYFIP1 were derived from the overexpression of AURKAIP1, we knockdown AURKAIP1 via siRNA (Fig. 8A). Indeed, AURKAIP1 knockdown partially reversed the anti-OS effects associated with CYFIP1 overexpression. After AURKAIP1 knockdown, FCM apoptosis assay shown that the apoptosis rate of OS cells significantly reduced while transwell and EdU assays found that OS cells migration ability was restored. (Fig. 8B-D). Meantime, flow cytometry assays also exhibited that the ROS level was decreased and MMP was elevated when AURKAIP1 was knockdown (Fig. 8E.F). Eventually, to validated whether the anti-OS effects of CYFIP1 could be attribute to cuproptosis, we utilized copper chelator to reverse the anti-OS effects of CYFIP1 and found that copper chelator could rescue OS cells apoptosis resulted from CYFIP1 overexpression (Fig. 8G). In the meantime, the application of copper chelator also partially counteract the inhibition derived from CYFIP1 overexpression on OS cells migration (Fig. 8H). Additionally, after the application of copper chelator, elevating ROS levels were weaken and MMP exhibited a downward trend, compared with DMSO group (Fig. 8I-J). These experimental results confirm that CYFIP1 interacts with RNMT to induce OS cuproptosis via the AURKAIP1/FDX1 pathway (Fig. 8K).

## Discussion

The advancement of treatments, particularly the introduction of multi-drug chemotherapies, has significantly extended the survival of OS patients(Gill and Gorlick 2021; Meltzer and Helman 2021). However, the survival rate for patients with metastatic and recurrent OS remains dismal(Ritter et al. 2010). Furthermore, the increasing development of chemotherapy resistance poses a challenge to improving the survival rate of OS patients(Meltzer and Helman 2021). Therefore, elucidating the underlying mechanisms of OS oncogenesis and progression is crucial for early diagnosis, precise treatment, and prognosis improvement. Recent studies have highlighted the therapeutic significance of epigenetics, in which m7G modification has garnered increasing attention due to substantial evidence indicating its impact on tumor pathogenesis and progression(Rong et al. 2021; Barbieri and Kouzarides 2020; Han et al. 2022, 2023; Bahr et al. 1999; Orellana et al. 2021). To date, m7G modification was proved to involve in the proliferation, migration, drug-resistance, apoptosis, and autophagy in certain cancers, such as lung, bladder, colon, pancreatic cancer and intrahepatic, esophageal squamous cell carcinoma(Luo et al. 2022; Xia et al. 2023; Pandolfini et al. 2019; Ma et al. 2021; Dai et al. 2021; Song et al. 2020). Therefore, understanding the effects and mechanisms of m7G modification contributes to the study of oncogenesis and progression in these cancers, providing novel insights into cancer treatments. Though studies conducted by Wang et al. revealed the importance of m7G modification in OS oncogenesis and progression, the function of m7G mRNA modification and its underlying mechanism in OS remain largely limited. In the present study, we first revealed the role and underlying mechanism of m7G modification derived from CYFIP1 and the tumor-suppressing role of CYFIP1 against OS. Mechanistically, we discovered that CYFIP1 could coordinate with RNMT to influence the m7G modification and facilitate the translational efficiency of AURKAIP1 mRNA, which lead to the upregulation of FDX1 and the subsequent OS cuproptosis, providing the researchers and clinicians with novel insight into OS researches and treatments.

In this study, through validated 90 OS patients in Xiangya hospital, we initially observed differential expression of CYFIP1 between normal tissues and OS tissues. Further exploration revealed a negative correlation between the expression of CYFIP1 and the prognosis of OS patients. Considering the differential expression and prognostic significance of CYFIP1, its role and mechanism may be crucial in the pathogenesis and development of OS. Previous research has shown that CYFIP1 can form binding complexes with eIF4E and FMR1 to interact with the m7G cap of mRNA, exerting a translation-suppressing effect(Rubeis et al. 2013; Bramham et al. 2016). Moreover, several recent studies have demonstrated the antitumor effects of CYFIP1 in various cancers(Chang et al. 2018; Silva et al. 2009; Dziunycz et al. 2017). However, it remains to be determined whether the effect of CYFIP1 on translation plays an anti-OS role. In our studies, we observed significant antitumor effects of CYFIP1, and mass spectrometry analysis indicated an interaction between CYFIP1 and RNMT. Earlier studies have identified RNMT as an RNA guanine-7 methyltransferase, primarily expressed in the nucleus and involved in mRNA cap methylation(Dunn et al. 2019; Aregger et al. 2016; Osborne et al. 2022). More recently, Trotman et al. discovered that RNMT is not only expressed in the nucleus but also in the cytoplasm, participating in the methylation of cytoplasmically recapped RNAs and regulating the translation of specific genes(Trotman et al. 2017). Additionally, Osborne et al. found that RNMT can compete for the binding of well-established eIF4E-binding proteins (4E-BPs) and form m7G cap-eIF4E-RNMT trimeric complexes, facilitating eIF4E to capture newly capped RNA(Osborne et al. 2022). Considering that CYFIP1 has been identified as a 4E-BP(Napoli et al. 2008), we further explored whether CYFIP1 interacts with RNMT to influence the translation of specific RNAs. Consistent with the findings of Trotman et al., our ICC analysis demonstrated the presence of RNMT in both the nucleus and cytoplasm, offering the possibility of interaction with CYFIP1, a cytoplasmic protein. Furthermore, co-IP further confirmed the interaction between CYFIP1 and RNMT. Collectively, considering that RNMT acts as an RNA guanine-7 methyltransferase, CYFIP1 may form complexes with RNMT to influence m7G modification in OS cells. As expected, the combination of m7G-RIP, RIP, and RNA stability assays confirmed that CYFIP1-RNMT complexes can m7G modify and stabilize AURKAIP1 mRNA, facilitating its translation. AURKAIP1, which is located in the mitochondrion and nucleoplasm, has been found to play a role in the positive regulation of proteolysis and is involved in mitochondrial translation and protein metabolism(Key et al. 2022; Koc et al. 2013).

Intriguingly, our transcriptome analysis revealed that besides translation-related pathways, there was also enrichment of mitochondrion translation-related pathways, indicating that changes in CYFIP1 expression might be associated with alterations in mitochondrial translation. To illuminate the effects derived from AURKAIP1, we performed the mitochondrial proteomics and found the upregulation of FDX1. Since cuproptosis was referred by Tsvetkov et al., cuproptosis-related genes, such as FDX1, gained enormous interest and were regarded as the emerging anticancer hotspots(Tsvetkov et al. 2022). Considering FDX1 serves as the core status of cuproptosis, we hypothesized that anti-OS effects of CYFIP1 may be associated with cuproptosis. Subsequently, our experimental results confirm that CYFIP1 overexpression resulted in MMP decline and elevated ROS level, leading to damaged mitochondrial structure and the eventual OS cuproptosis. Eventually, we knock downed AURKAIP1 and utilized copper chelator to recuse the anti-OS effects derived from CYFIP1 overexpression and found AURKAIP1 knockdown and copper chelator could reverse the pro-apoptosis, elevating ROS level, and MMP decline resulted from CYFIP1 overexpression. Taken altogether, CYFIP1 could coordinate with RNMT to trigger OS cuproptosis through m7G modifying AURKAIP1 mRNA. Previous studies highlighted CYFIP1 served as the member of WRC complexes and CYFIP1-EIF4E-FMR1 complexes, broadly involving in the cytoskeletal dynamics and protein translation of various cancers(Rubeis et al. 2013; Marino et al. 2015; Teng et al. 2016a, b; Silva et al. 2009). Here, we revealed the role of CYFIP1 during m7G modification and cuproptosis, providing new perspective into the role and mechanism of CYFIP1.

Since the genome complexity and instability of OS have baffled the targeted therapies, OS treatments fail to achieve revolutionary progresses(Wu and Livingston 2020; Kansara et al. 2014). Though classical pathways, such as PTEN/PI3K/AKT, VEGF/VEGFR pathways, were found to involve in certain OS occurrence and progression, the intervening measure targeting these pathways fail to benefit all OS patients, indicating an urgency to seek the common targets of OS patients(Gill and Gorlick 2021). All living organisms, especially cancer cells, need to undergo energy metabolism, so targeting energy metabolism may be a viable approach for OS treatments(Porporato et al. 2018; Pavlova et al. 2022; Xia et al. 2021). Cuproptosis serves as an emerging cell death type targeting TCA cycle(Tsvetkov et al. 2022) and increasing evidence demonstrates that the TCA cycle is essential for the energy production and macromolecule synthesis of certain cancer cells with dysregulated oncogene and tumor suppressor expression(Anderson et al. 2018; Eniafe and Jiang 2021). Our study revealed the role of CYFIP1 in m7G modification and cuproptosis in OS, which ultimately triggers cuproptosis in OS cells, thereby suppressing the occurrence and progression of OS. Therefore, intervening in key targets of cuproptosis, which in turn interfere with energy metabolism and macromolecular synthesis, may be a feasible treatment method for OS. Additionally, given that the energy metabolism of cancer cells can be interconverted between glycolysis and oxidative phosphorylation(Pavlova et al. 2022), targeting the TCA cycle with the cuproptosis inducers might be utilized along with the glycolysis inhibitors to achieve more complete anticancer inhibition effects in the treatment of OS. This offers more potential drug combination regimens for the clinicians.

In conclusion, our study demonstrates that CYFIP1 acts as a potent tumor suppressor in OS. The overexpression of CYFIP1 significantly inhibits OS cell proliferation and metastasis, while promoting apoptosis. Mechanistically, CYFIP1, in coordination with RNMT, stabilizes AURKAIP1 mRNA and enhances its translation, leading to the upregulation of FDX1 and the consequent OS cells cuproptosis. These findings provide a solid foundation for further research and potential therapeutic applications involving m7G modification and cuproptosis in the context of OS.

## Electronic supplementary material

Below is the link to the electronic supplementary material.

Supplementary Material 1

Supplementary Material 2

## Data Availability

No datasets were generated or analysed during the current study.

## Abbreviations

OS: Osteosarcoma
m7G: N7-methylguanosine
CYFIP1: Cytoplasmic FMR1 Interacting Protein 1
WRC: WAVE regulatory complex
TEM: Transmission electron microscope
MMP: Mitochondrial membrane potential
ROS: Reactive oxygen species
TCA: Tricarboxylic acid
PPI: Protein-protein interaction
GSEA: Gene set enrichment analysis
KEGG: Kyoto Encyclopedia of Genes and Genomes
GO: Gene Ontology
DEGs: Differentially expressed genes
IHC: Immunohistochemistry
ICC: Immunocytochemistry
qPCR: Quantitative polymerase chain reaction
FDR: false discovery rate

## References

[CR36] Abekhoukh S, Bardoni B. CYFIP family proteins between autism and intellectual disability: links with Fragile X syndrome. Front Cell Neurosci. 2014;8:81. 10.3389/fncel.2014.00081. 24733999 10.3389/fncel.2014.00081 PMC3973919

[CR33] Abekhoukh S, et al. New insights into the regulatory function of CYFIP1 in the context of WAVE- and FMRP-containing complexes. Dis Models Mech. 2017;10:463–74. 10.1242/dmm.025809. 10.1242/dmm.025809 PMC5399562 28183735

[CR29] Alexandrov A, Martzen MR, Phizicky EM. Two proteins that form a complex are required for 7-methylguanosine modification of yeast tRNA. RNA (New York N Y). 2002;8:1253–66. 10.1017/s1355838202024019. 12403464 10.1017/s1355838202024019 PMC1370335

[CR66] Anderson NM, Mucka P, Kern JG, Feng H. The emerging role and targetability of the TCA cycle in cancer metabolism. Protein Cell. 2018;9:216–37. 10.1007/s13238-017-0451-1. 28748451 10.1007/s13238-017-0451-1 PMC5818369

[CR24] Arbour KC, et al. Treatment outcomes and clinical characteristics of patients with KRAS-G12C-Mutant Non-small Cell Lung Cancer. Clin cancer Research: Official J Am Association Cancer Res. 2021;27:2209–15. 10.1158/1078-0432.Ccr-20-4023. 10.1158/1078-0432.CCR-20-4023 PMC8771577 33558425

[CR57] Aregger M, et al. CDK1-Cyclin B1 activates RNMT, coordinating mRNA Cap methylation with G1 phase transcription. Mol Cell. 2016;61:734–46. 10.1016/j.molcel.2016.02.008. 26942677 10.1016/j.molcel.2016.02.008 PMC4781437

[CR49] Bahr A, Hankeln T, Fiedler T, Hegemann J, Schmidt ER. Molecular analysis of METTL1, a novel human methyltransferase-like gene with a high degree of phylogenetic conservation. Genomics. 1999;57:424–8. 10.1006/geno.1999.5780. 10329009 10.1006/geno.1999.5780

[CR47] Barbieri I, Kouzarides T. Role of RNA modifications in cancer. Nat Rev Cancer. 2020;20:303–22. 10.1038/s41568-020-0253-2. 32300195 10.1038/s41568-020-0253-2

[CR37] Bonaccorso CM et al. Fragile X mental retardation protein (FMRP) interacting proteins exhibit different expression patterns during development. 42, 15–23, 10.1016/j.ijdevneu.2015.02.004 (2015). 10.1016/j.ijdevneu.2015.02.004 25681562

[CR55] Bramham CR, Jensen KB, Proud CG. Tuning specific translation in Cancer metastasis and synaptic memory: control at the MNK-eIF4E Axis. Trends Biochem Sci. 2016;41:847–58. 10.1016/j.tibs.2016.07.008. 27527252 10.1016/j.tibs.2016.07.008

[CR40] Chang JW, et al. Wild-type p53 upregulates an early onset breast cancer-associated gene GAS7 to suppress metastasis via GAS7-CYFIP1-mediated signaling pathway. Oncogene. 2018;37:4137–50. 10.1038/s41388-018-0253-9. 29706651 10.1038/s41388-018-0253-9 PMC6062498

[CR28] Chen Z, et al. METTL1 promotes hepatocarcinogenesis via m(7) G tRNA modification-dependent translation control. Clin Translational Med. 2021;11(e661). 10.1002/ctm2.661. 10.1002/ctm2.661 PMC8666584 34898034

[CR27] Chu C, Shatkin AJ. Apoptosis and autophagy induction in mammalian cells by small interfering RNA knockdown of mRNA capping enzymes. Mol Cell Biol. 2008;28:5829–36. 10.1128/mcb.00021-08. 18678651 10.1128/MCB.00021-08 PMC2547012

[CR53] Dai Z, et al. N(7)-Methylguanosine tRNA modification enhances oncogenic mRNA translation and promotes intrahepatic cholangiocarcinoma progression. Mol Cell. 2021;81:3339–e33553338. 10.1016/j.molcel.2021.07.003. 34352206 10.1016/j.molcel.2021.07.003

[CR32] De Rubeis S, et al. CYFIP1 coordinates mRNA translation and cytoskeleton remodeling to ensure proper dendritic spine formation. Neuron. 2013;79:1169–82. 10.1016/j.neuron.2013.06.039. 24050404 10.1016/j.neuron.2013.06.039 PMC3781321

[CR34] Di Marino D, et al. MD and Docking studies Reveal that the Functional switch of CYFIP1 is mediated by a Butterfly-like motion. J Chem Theory Comput. 2015;11:3401–10. 10.1021/ct500431h. 26575774 10.1021/ct500431h

[CR19] Drummond DR, Armstrong J, Colman A. The effect of capping and polyadenylation on the stability, movement and translation of synthetic messenger RNAs in Xenopus oocytes. Nucleic Acids Res. 1985;13:7375–94. 10.1093/nar/13.20.7375. 3932972 10.1093/nar/13.20.7375 PMC322050

[CR26] Dunn S, Lombardi O, Lukoszek R, Cowling VH. Oncogenic PIK3CA mutations increase dependency on the mRNA cap methyltransferase, RNMT, in breast cancer cells. Open Biology. 2019;9:190052. 10.1098/rsob.190052. 30991934 10.1098/rsob.190052 PMC6501644

[CR56] Dziunycz PJ, et al. CYFIP1 is directly controlled by NOTCH1 and down-regulated in cutaneous squamous cell carcinoma. PLoS ONE. 2017;12:e0173000. 10.1371/journal.pone.0173000. 28410392 10.1371/journal.pone.0173000 PMC5391925

[CR67] Eniafe J, Jiang S. The functional roles of TCA cycle metabolites in cancer. Oncogene. 2021;40:3351–63. 10.1038/s41388-020-01639-8. 33864000 10.1038/s41388-020-01639-8

[CR6] Fresquet V, et al. Endogenous retroelement activation by epigenetic therapy reverses the Warburg Effect and elicits mitochondrial-mediated Cancer cell death. Cancer Discov. 2021;11:1268–85. 10.1158/2159-8290.Cd-20-1065. 33355179 10.1158/2159-8290.CD-20-1065

[CR21] Furuichi Y, LaFiandra A, Shatkin AJ. 5’-Terminal structure and mRNA stability. Nature. 1977;266:235–9. 10.1038/266235a0. 557727 10.1038/266235a0

[CR2] Gill J, Gorlick R. Advancing therapy for osteosarcoma. Nat Rev Clin Oncol. 2021;18:609–24. 10.1038/s41571-021-00519-8. 34131316 10.1038/s41571-021-00519-8

[CR48] Han H, et al. N(7)-methylguanosine tRNA modification promotes esophageal squamous cell carcinoma tumorigenesis via the RPTOR/ULK1/autophagy axis. Nat Commun. 2022;13:1478. 10.1038/s41467-022-29125-7. 35304469 10.1038/s41467-022-29125-7 PMC8933395

[CR50] Han H, Zheng S, Lin S. N(7)-methylguanosine (m(7)G) tRNA modification: a novel autophagy modulator in cancer. Autophagy. 2023;19:360–2. 10.1080/15548627.2022.2077551. 35574843 10.1080/15548627.2022.2077551 PMC9809925

[CR31] He M, et al. M(7)G modification of FTH1 and pri-miR-26a regulates ferroptosis and chemotherapy resistance in osteosarcoma. Oncogene. 2024;43:341–53. 10.1038/s41388-023-02882-5. 38040806 10.1038/s41388-023-02882-5

[CR5] Kansara M, Teng MW, Smyth MJ, Thomas DM. Translational biology of osteosarcoma. Nat Rev Cancer. 2014;14:722–35. 10.1038/nrc3838. 25319867 10.1038/nrc3838

[CR60] Key J, et al. CLPP depletion causes Diplotene arrest; underlying Testis mitochondrial dysfunction occurs with Accumulation of Perrault Proteins ERAL1, PEO1, and HARS2. Cells. 2022;12. 10.3390/cells12010052. 10.3390/cells12010052 PMC9818230 36611846

[CR61] Koc EC, et al. Identification and characterization of CHCHD1, AURKAIP1, and CRIF1 as new members of the mammalian mitochondrial ribosome. Front Physiol. 2013;4:183. 10.3389/fphys.2013.00183. 23908630 10.3389/fphys.2013.00183 PMC3726836

[CR17] Konarska MM, Padgett RA, Sharp PA. Recognition of cap structure in splicing in vitro of mRNA precursors. Cell. 1984;38:731–6. 10.1016/0092-8674(84)90268-x. 6567484 10.1016/0092-8674(84)90268-x

[CR20] Lewis JD, Izaurralde E. The role of the cap structure in RNA processing and nuclear export. Eur J Biochem. 1997;247:461–9. 10.1111/j.1432-1033.1997.00461.x. 9266685 10.1111/j.1432-1033.1997.00461.x

[CR41] Limaye AJ, Whittaker MK, Bendzunas GN, Cowell JK, Kennedy EJ. Targeting the WASF3 complex to suppress metastasis. Pharmacol Res. 2022;182:106302. 10.1016/j.phrs.2022.106302. 35691539 10.1016/j.phrs.2022.106302 PMC12392151

[CR18] Lindstrom DL, et al. Dual roles for Spt5 in pre-mRNA processing and transcription elongation revealed by identification of Spt5-associated proteins. Mol Cell Biol. 2003;23:1368–78. 10.1128/mcb.23.4.1368-1378.2003. 12556496 10.1128/MCB.23.4.1368-1378.2003 PMC141151

[CR44] Love MI, Huber W, Anders S. Moderated estimation of Fold change and dispersion for RNA-seq data with DESeq2. Genome Biol. 2014;15(550). 10.1186/s13059-014-0550-8. 10.1186/s13059-014-0550-8 PMC4302049 25516281

[CR11] Luo Y, et al. The potential role of N(7)-methylguanosine (m7G) in cancer. J Hematol Oncol. 2022;15. 10.1186/s13045-022-01285-5. 10.1186/s13045-022-01285-5 PMC9118743 35590385

[CR52] Ma J, et al. METTL1/WDR4-mediated m(7)G tRNA modifications and m(7)G codon usage promote mRNA translation and lung cancer progression. Mol Therapy: J Am Soc Gene Therapy. 2021;29:3422–35. 10.1016/j.ymthe.2021.08.005. 10.1016/j.ymthe.2021.08.005 PMC8636169 34371184

[CR3] Meltzer PS, Helman LJ. New Horizons in the Treatment of Osteosarcoma. N Engl J Med. 2021;385:2066–76. 10.1056/NEJMra2103423. 34818481 10.1056/NEJMra2103423

[CR10] Merrick WC, Pavitt GD. Protein Synthesis Initiation in Eukaryotic Cells. *Cold Spring Harbor perspectives in biology* 10. 10.1101/cshperspect.a033092 (2018). 10.1101/cshperspect.a033092 PMC6280705 29735639

[CR15] Murthy KG, Park P, Manley JL. A nuclear micrococcal-sensitive, ATP-dependent exoribonuclease degrades uncapped but not capped RNA substrates. Nucleic Acids Res. 1991;19:2685–92. 10.1093/nar/19.10.2685. 1710342 10.1093/nar/19.10.2685 PMC328187

[CR13] Muthukrishnan S, Both GW, Furuichi Y, Shatkin AJ. 5’-Terminal 7-methylguanosine in eukaryotic mRNA is required for translation. Nature. 1975;255:33–7. 10.1038/255033a0. 165427 10.1038/255033a0

[CR35] Napoli I, et al. The fragile X syndrome protein represses activity-dependent translation through CYFIP1, a new 4E-BP. Cell. 2008;134:1042–54. 10.1016/j.cell.2008.07.031. 18805096 10.1016/j.cell.2008.07.031

[CR51] Orellana EA, et al. METTL1-mediated m(7)G modification of Arg-TCT tRNA drives oncogenic transformation. Mol Cell. 2021;81:3323–e33383314. 10.1016/j.molcel.2021.06.031. 34352207 10.1016/j.molcel.2021.06.031 PMC8380730

[CR58] Osborne MJ, et al. Identification and characterization of the Interaction between the Methyl-7-Guanosine Cap Maturation enzyme RNMT and the Cap-binding protein eIF4E. J Mol Biol. 2022;434:167451. 10.1016/j.jmb.2022.167451. 35026230 10.1016/j.jmb.2022.167451 PMC9288840

[CR22] Pandolfini L, et al. METTL1 promotes let-7 MicroRNA Processing via m7G methylation. Mol Cell. 2019;74:1278–e12901279. 10.1016/j.molcel.2019.03.040. 31031083 10.1016/j.molcel.2019.03.040 PMC6591002

[CR64] Pavlova NN, Zhu J, Thompson CB. The hallmarks of cancer metabolism: still emerging. Cell Metabol. 2022;34:355–77. 10.1016/j.cmet.2022.01.007. 10.1016/j.cmet.2022.01.007 PMC8891094 35123658

[CR16] Pei Y, Shuman S. Interactions between fission yeast mRNA capping enzymes and elongation factor Spt5. J Biol Chem. 2002;277:19639–48. 10.1074/jbc.M200015200. 11893740 10.1074/jbc.M200015200

[CR63] Porporato PE, Filigheddu N, Pedro JMB, Kroemer G, Galluzzi L. Mitochondrial metabolism and cancer. Cell Res. 2018;28:265–80. 10.1038/cr.2017.155. 29219147 10.1038/cr.2017.155 PMC5835768

[CR1] Ritter J, Bielack SS, Osteosarcoma. Annals Oncology: Official J Eur Soc Med Oncol. 2010;21. 10.1093/annonc/mdq276. Suppl 7, vii320-325. 10.1093/annonc/mdq276 20943636

[CR46] Rong D, et al. Epigenetics: roles and therapeutic implications of non-coding RNA modifications in human cancers. Mol Therapy Nucleic Acids. 2021;25:67–82. 10.1016/j.omtn.2021.04.021. 34188972 10.1016/j.omtn.2021.04.021 PMC8217334

[CR8] Roundtree IA, Evans ME, Pan T, He C. Dynamic RNA modifications in Gene expression regulation. Cell. 2017;169:1187–200. 10.1016/j.cell.2017.05.045. 28622506 10.1016/j.cell.2017.05.045 PMC5657247

[CR14] Shimotohno K, Kodama Y, Hashimoto J, Miura KI. Importance of 5’-terminal blocking structure to stabilize mRNA in eukaryotic protein synthesis. Proc Natl Acad Sci USA. 1977;74:2734–8. 10.1073/pnas.74.7.2734. 197518 10.1073/pnas.74.7.2734 PMC431268

[CR42] Silva JM, et al. Cyfip1 is a putative invasion suppressor in epithelial cancers. Cell. 2009;137:1047–61. 10.1016/j.cell.2009.04.013. 19524508 10.1016/j.cell.2009.04.013 PMC2754270

[CR54] Song B, et al. m7GHub: deciphering the location, regulation and pathogenesis of internal mRNA N7-methylguanosine (m7G) sites in human. Bioinf (Oxford England). 2020;36:3528–36. 10.1093/bioinformatics/btaa178. 10.1093/bioinformatics/btaa178 32163126

[CR25] Stefanska B, et al. Genome-wide study of hypomethylated and induced genes in patients with liver cancer unravels novel anticancer targets. Clin cancer Research: Official J Am Association Cancer Res. 2014;20:3118–32. 10.1158/1078-0432.Ccr-13-0283. 10.1158/1078-0432.CCR-13-0283 24763612

[CR7] Tang M, et al. Epigenetic induction of mitochondrial fission is required for maintenance of Liver Cancer-initiating cells. Cancer Res. 2021;81:3835–48. 10.1158/0008-5472.Can-21-0436. 34049973 10.1158/0008-5472.CAN-21-0436

[CR38] Teng Y, et al. The WASF3-NCKAP1-CYFIP1 complex is essential for breast Cancer metastasis. Cancer Res. 2016a;76:5133–42. 10.1158/0008-5472.Can-16-0562. 27432794 10.1158/0008-5472.CAN-16-0562 PMC5010469

[CR39] Teng Y, et al. Targeting the WASF3-CYFIP1 complex using stapled peptides suppresses Cancer Cell Invasion. Cancer Res. 2016b;76:965–73. 10.1158/0008-5472.Can-15-1680. 26676744 10.1158/0008-5472.CAN-15-1680 PMC4828974

[CR23] Tian QH, et al. METTL1 overexpression is correlated with poor prognosis and promotes hepatocellular carcinoma via PTEN. J Mol Med. 2019;97:1535–45. 10.1007/s00109-019-01830-9. 31463732 10.1007/s00109-019-01830-9

[CR59] Trotman JB, Giltmier AJ, Mukherjee C, Schoenberg DR. RNA guanine-7 methyltransferase catalyzes the methylation of cytoplasmically recapped RNAs. Nucleic Acids Res. 2017;45:10726–39. 10.1093/nar/gkx801. 28981715 10.1093/nar/gkx801 PMC5737702

[CR62] Tsvetkov P, et al. Copper induces cell death by targeting lipoylated TCA cycle proteins. Sci (New York N Y). 2022;375:1254–61. 10.1126/science.abf0529. 10.1126/science.abf0529 PMC9273333 35298263

[CR30] Wang Z, et al. METTL1/WDR4-mediated tRNA m(7)G modification and mRNA translation control promote oncogenesis and doxorubicin resistance. Oncogene. 2023;42:1900–12. 10.1038/s41388-023-02695-6. 37185458 10.1038/s41388-023-02695-6

[CR4] Wu CC, Livingston JA. Genomics and the Immune Landscape of Osteosarcoma. Adv Exp Med Biol. 2020;1258:21–36. 10.1007/978-3-030-43085-6_2. 32767232 10.1007/978-3-030-43085-6_2

[CR65] Xia L, et al. The cancer metabolic reprogramming and immune response. Mol Cancer. 2021;20:28. 10.1186/s12943-021-01316-8. 33546704 10.1186/s12943-021-01316-8 PMC7863491

[CR12] Xia X, Wang Y, Zheng JC. Internal m7G methylation: a novel epitranscriptomic contributor in brain development and diseases. Mol Therapy Nucleic Acids. 2023;31:295–308. 10.1016/j.omtn.2023.01.003. 36726408 10.1016/j.omtn.2023.01.003 PMC9883147

[CR45] Xiao C, et al. Arabidopsis DXO1 activates RNMT1 to methylate the mRNA guanosine cap. Nat Commun. 2023;14:202. 10.1038/s41467-023-35903-8. 36639378 10.1038/s41467-023-35903-8 PMC9839713

[CR43] Yuan Y, et al. TXNIP inhibits the progression of osteosarcoma through DDIT4-mediated mTORC1 suppression. Am J cancer Res. 2022;12:3760–79. 36119812 PMC9442022

[CR9] Zhao B, Roundtree I, He CJ. N. r. M. c. b. post-transcriptional gene regulation by mRNA modifications. 18, 31–42, 10.1038/nrm.2016.132 (2017). 10.1038/nrm.2016.132 PMC5167638 27808276

