# Correction: CYFIP1 coordinate with RNMT to induce osteosarcoma cuproptosis via AURKAIP1 m7G modification

**DOI:** 10.1186/s10020-025-01185-7

**Published:** 2025-04-07

**Authors:** Zili Lin, Ziyi Wu, Yizhe He, Xiangyao Li, Wei Luo

**Affiliations:** 1 Department of Orthopaedics, Xiangya Hospital, Central South University, Changsha, 410008 Hunan People’s Republic of China; 2 National Clinical Research Center for Geriatric Disorders, Xiangya Hospital, Changsha, 410008 Hunan People’s Republic of China; 3 Department of Orthopaedics, The Second Xiangya Hospital, Central South University, Changsha, 410011 Hunan People’s Republic of China

**Correction** **:** **Molecular Medicine (2025) 31:74** 10.1186/s10020-025-01127-3

In this article (Lin et al. 2025), Fig. 3 appeared incorrectly and have now been corrected in the original publication. For completeness and transparency, both incorrect and correct versions are displayed below.

The original article has been corrected.

Incorrect Fig. 3
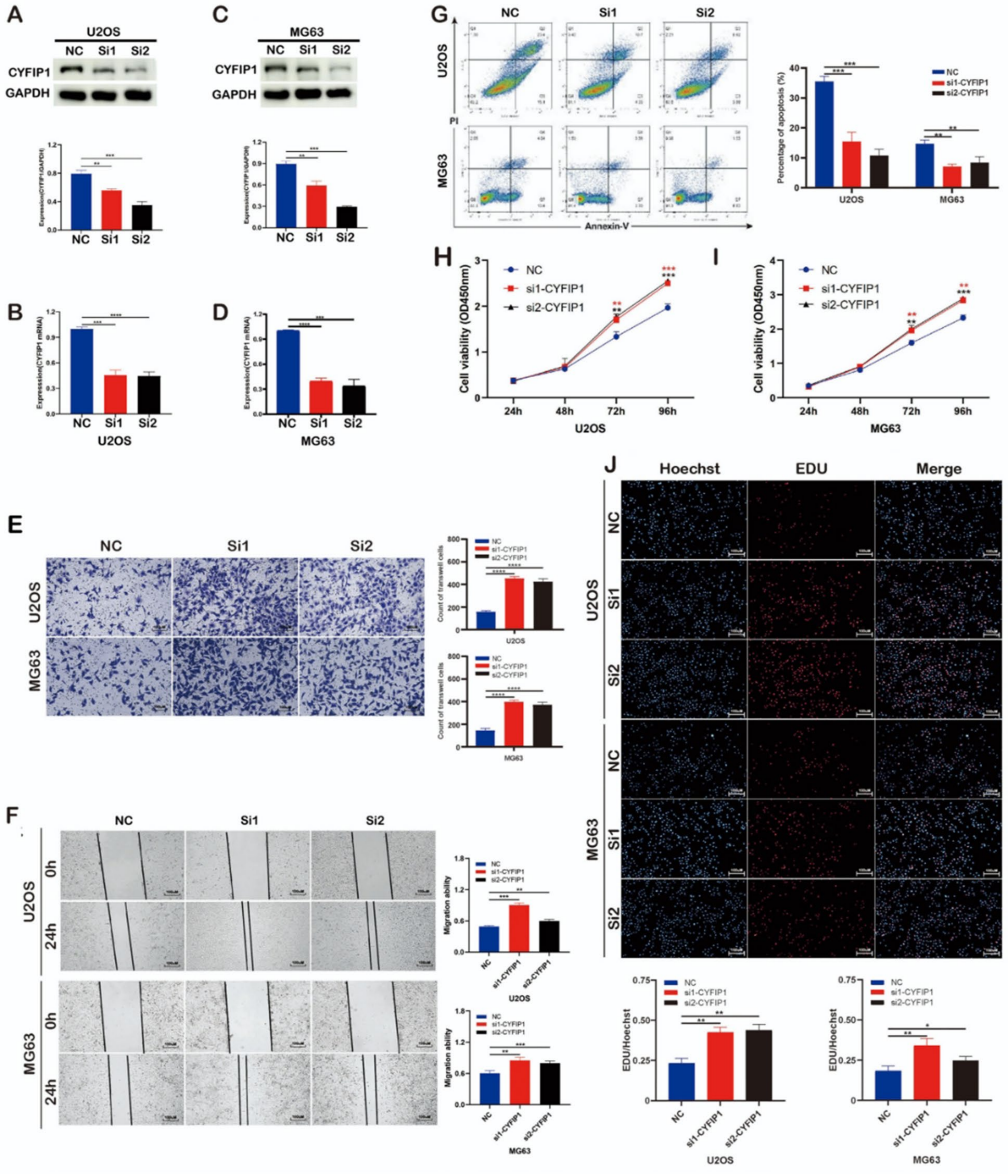


Correct Fig. 3

**Fig. 3:**
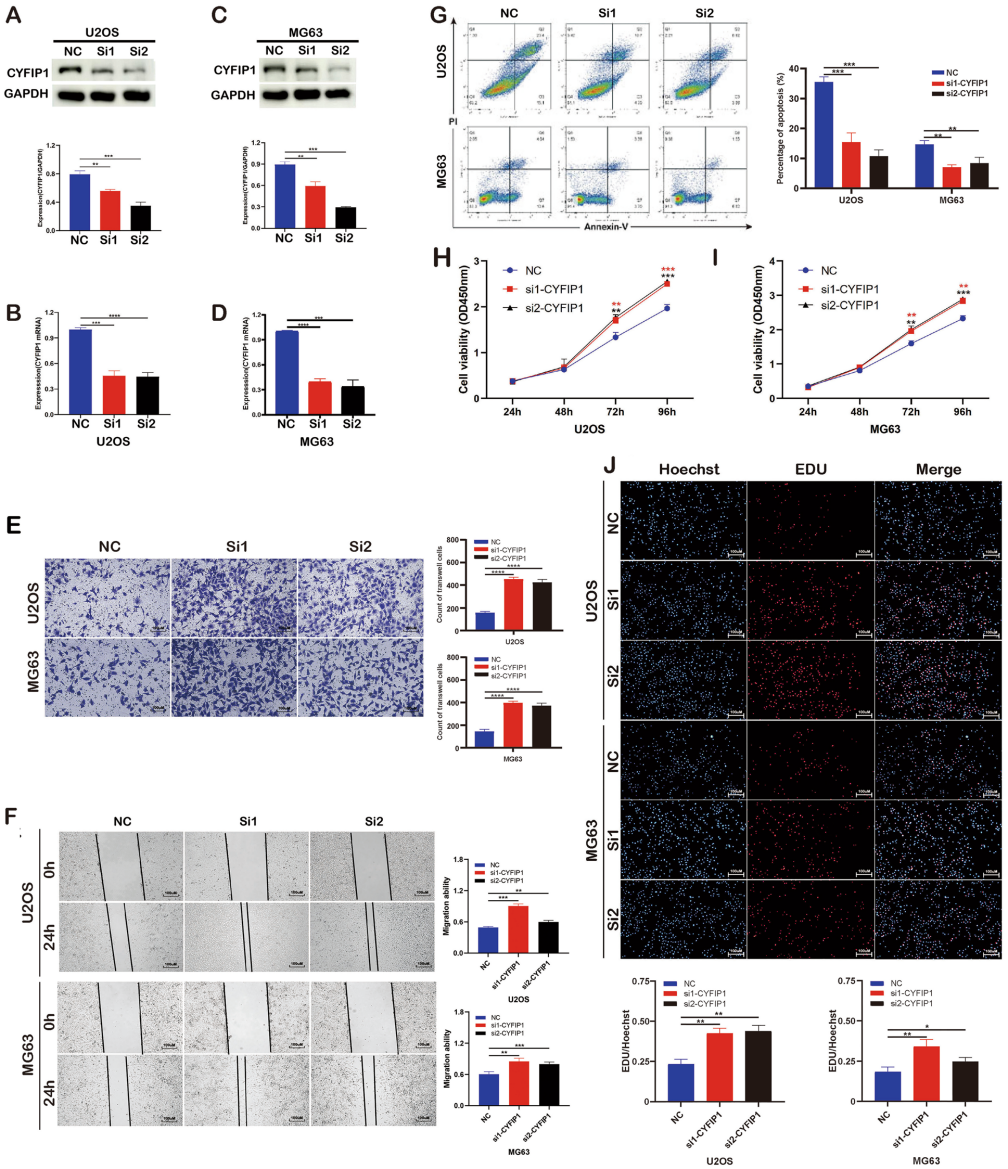
Validation of the role of CYFIP1 via downregulation experiment. **A–D** WB and qPCR exhibited the knockdown efficiency of shRNA transfection. **E**, **F** Knockdown of CYFIP1 promoted osteosarcoma cells migration. **G** Knockdown of CYFIP1 decreased osteosarcoma cells apoptosis. **H–J** CCK8 and EdU assays exhibited knockdown of CYFIP1 promoted osteosarcoma cells proliferation. The data represent the mean ± S.D. *P < 0.05, **P < 0.01, ***P < 0.001, and ****P < 0.0001 indicates a significant difference between the indicated groups
