# Supplementary material for: CYFIP1 coordinate with RNMT to induce osteosarcoma cuproptosis via AURKAIP1 m7G modification
Source: Mol Med. 2025 Feb 21;31:74. doi: 10.1186/s10020-025-01127-3 (PMC11846196; doi:10.1186/s10020-025-01127-3)
Supplement: Supplementary file 1 — Supplementary Material 1 [file 10020_2025_1127_MOESM1_ESM.docx]

| Supplementary table1 |  |
| --- | --- |
| siRNA or shRNA | Sequence |
| shCYFIP1_1 | GCTGCTCTACCAGCCAAATTT |
| shCYFIP1_2 | GCAGCCCTCAGTACAACATCT |
| siAURKAIP1 | GGATGCGCCTCAAATTCAGTGCAAA |
| siRNMT | AAGAGTTTGAAGATGATCTTGTAAA |

Supplementary table2

| Primer | Sequence |
| --- | --- |
| CYFIP1_F | CTGCACGCGGCTCCTTTCCA |
| CYFIP1_R | GACAAGATGCAGCGGGGCGT |
| UBR4_F | GTCCGTCTAATGCTGTTGG |
| UBR4_R | TCGTAATTGAGGCAGGGT |
| NCEH1_F | TGGTGTTGCCTAGATCCC |
| NCEH1_R | GCTTGGGCTGTTGAAACT |
| TK1_F | CTGCACTGGATGGGACCT |
| TK1_R | GCTTCACCACGCTCTCG |
| GPRC5C_F | GGGCAAGGGACTCTCCA |
| GPRC5C_R | ATGACACCCACCCAAACAC |
| AURKAIP1_F | GCTGGAGCTGGAGGAGATG |
| AURKAIP1_R | TGGTAGGATTGCGGTGGA |
| C16orf72_F | TCCCGAGTGAGAAGATGG |
| C16orf72_R | AGTGAAAGTGGGGCTTTG |
| PYCR1_F | ACCCAGGCCAGCTCAAG |
| PYCR1_R | CCCCACTCTCCAGCACA |
| GPANK1_F | GCCCCTAGTGCGAATCAG |
| GPANK1_R | GAGCCCGGAGGAAACAG |
| CHD8_F | GGTCATCACACTGAAACGG |
| CHD8_R | TTAGAAAGGTCTGGTCGCA |

Supplementary figure1 (A)
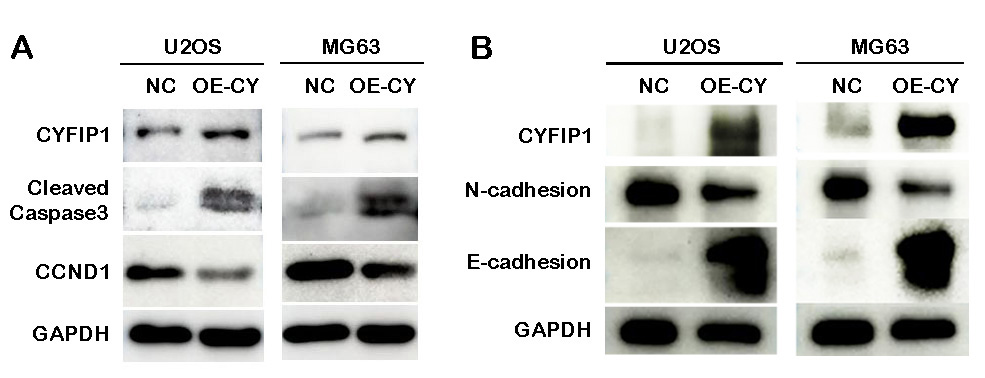
 The overexpression of CYFIP1 could increase Cleaved Caspase-3 expression but decrease CCND1 expression. (B) The overexpression of CYFIP1 could increase E-cadhesion expression but decrease N-cadhesion expression.


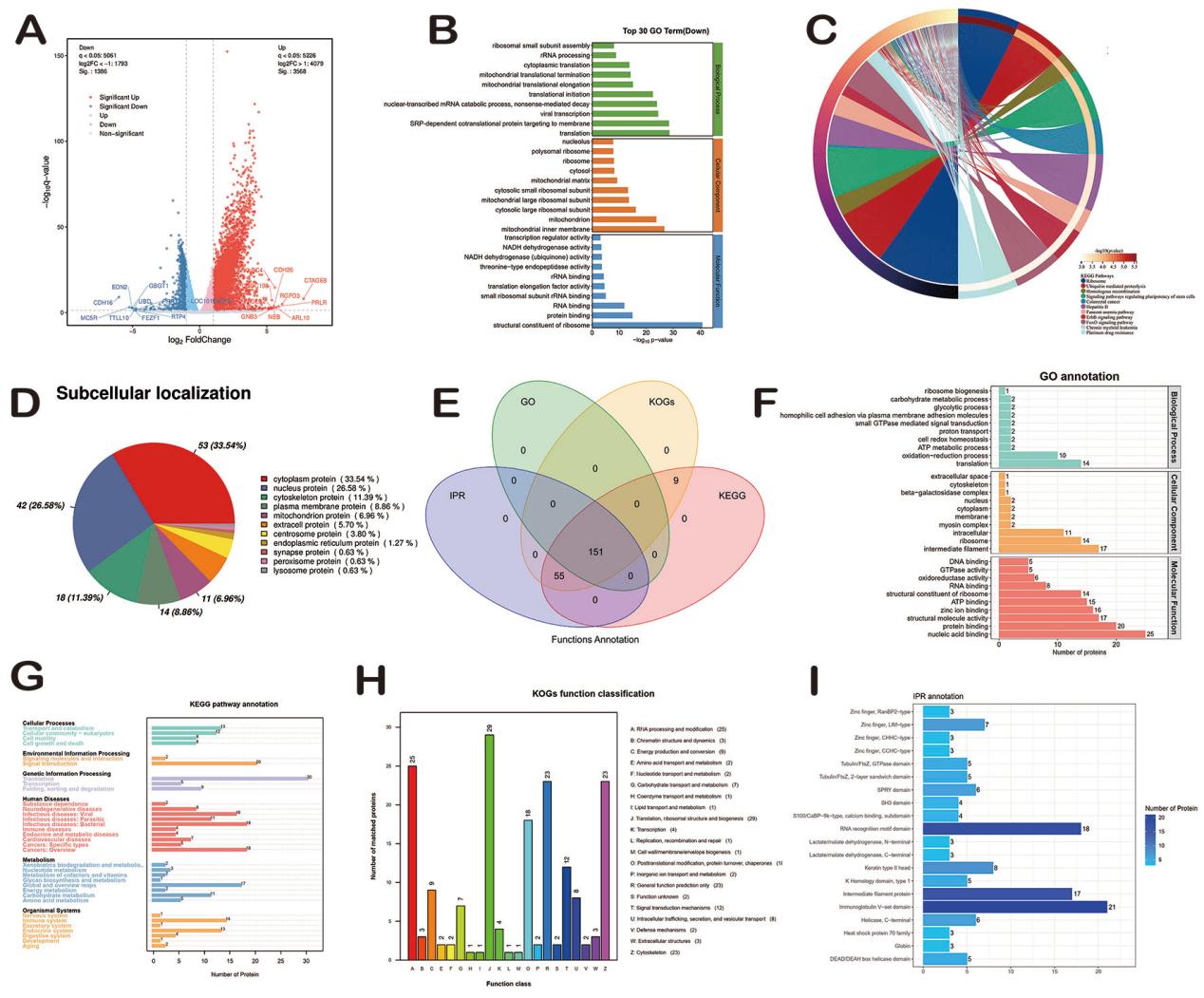


Supplementary figure2 (A) The Volcano plot of RNA seqencing between OE-CYFIP1 and NC groups. (B) GO enrichment analysis of DEGs in RNA seqencing. (C) KEGG enrichment analysis of DEGs in RNA seqencing. (D)Subcellular localization of CYFIP1-interacting protein identified by mass spectrum. (E) Venn plot of GO, KEGG, KOGs, and IPR analyses. (F) GO analysis regarding CYFIP1-interacting proteins in mass spectrum. (G) KEGG analysis regarding CYFIP1-interacting proteins in mass spectrum. (H) KOGs analysis regarding CYFIP1-interacting proteins in mass spectrum. (I) IPR analysis regarding CYFIP1-interacting proteins in mass spectrum.


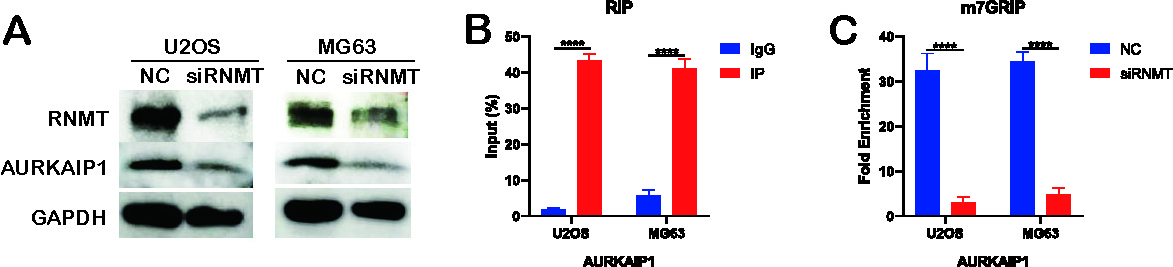


Supplementary figure3 (A) Western blotting to detect the effect of RNMT knockdown on AURKAIP1 expression. (B) RIP assay. (C) m7G-MeRIP assay.
